# Proanthocyanidins in immune-mediated inflammatory diseases: molecular mechanisms and therapeutic potential

**DOI:** 10.3389/fphar.2026.1958407

**Published:** 2026-09-25

**Authors:** Yu Yang, Ke Xian, Rui Lai, Li Yang, Jian Qiao Zhong

**Affiliations:** 1 Department of Dermatology, The Affiliated Hospital of Southwest Medical University, Luzhou, China; 2 Clinical Medical College, Southwest Medical University, Luzhou, China; 3 Department of Dermatology, The First People’s Hospital of Yibin, Yibin, China; 4 Department of Dermatology, Xuyong People’s Hospital, Xuyong, China

**Keywords:** atopic dermatitis, crohn's disease, immue-mediated inflammatory diseases, proanthocyanidins, psoriasis, ulcerative colitis

## Abstract

Immune-mediated inflammatory diseases (IMIDs), a heterogeneous group of disorders, are characterized by chronic inflammation and subsequent organ damage, encompassing diverse conditions like atopic dermatitis (AD), inflammatory bowel disease (IBD) - which includes Crohn’s disease (CD) and ulcerative colitis (UC), psoriasis, rheumatoid arthritis (RA), and systemic lupus erythematosus (SLE). These chronic diseases mainly stem from inappropriate or excessive immune responses. In recent years, the incidence of IMIDs has been increasing. Considering their frequent association with various complications, such as cardiovascular diseases, cognitive impairment, metabolic disorders, and high-level disabilities, IMIDs have emerged as a major medical challenge. Proanthocyanidins (PCs), also known as condensed tannins, are among the most abundant polyphenols in the human diet. Owing to their health-protective activities, they have attracted extensive research interest. These compounds are commonly found in plants, including fruits, nuts, bark, chocolate, wine, as well as some plant seeds and flowers. A multitude of studies have demonstrated that PCs possess a wide range of pharmacological properties, such as antioxidant effects, anti-inflammatory action, immune regulation, cardiovascular benefits, and antineoplastic effects. It has been demonstrated that PCs, as natural plant compounds, are safe and rare of notable adverse effects. Their extensive range of biological activities has facilitated their applications in treating various IMIDs. In this review, we provide a comprehensive overview of the diverse biological activities of PCs and their favorable roles in common IMIDs, while also elucidating the underlying mechanisms involved in IMIDs.

## 1 Introduction

Immue-mediated inflammatory diseases (IMIDs) are a group of chronic inflammatory diseases caused by abnormal activation or dysregulation of the immune system (Scherlinger et al., 2023). These disorders share key characteristics in their clinical manifestation, target organs, and long-term outcomes. Their core pathogenesis lies in the aberrant activation of the body’s immune response, which subsequently triggers a self-destructive process causing damage to host tissues (Kuek et al., 2007; Belling et al., 2018). Emerging evidence indicates that the overactivation of T-helper 17 (Th17) cells and the release of their pro-inflammatory cytokines, mainly interleukin-17A (IL-17A) and interleukin-17F (IL-17F), propel the inflammatory cascade central to IMIDs (Monteleone et al., 2023). Moreover, immune imbalance resulting from reduced numbers or functional impairment of regulatory T (Treg) cells contributes significantly to the development of these diseases (Kerdidani et al., 2022). Notably, cytokines including tumor necrosis factor-α (TNF-α) and interleukin-23 (IL-23) are central drivers of the pathophysiological network underlying IMIDs (Sachen et al., 2025). Additionally, gut microbiota dysbiosis, as an influential environmental factor, fuels both the initiation and progression of diseases through disrupting intestinal barrier integrity, provoking immune responses, and releasing immunomodulatory metabolites (Monteleone et al., 2023). The above inflammatory factors and their signaling cascades across multiple tissue systems, ultimately converging into a spectrum of diseases with diverse clinical presentations. In daily practice, IMIDs are still sorted by the simplest possible rubric: the site of injury. Clinically, IMIDs are most concisely grouped basing on the affected organs, just as shown in Table 1 (Monteleone et al., 2023; Thomas et al., 2024; Global and regional, 2023; Bladé et al., 2016; Rauf et al., 2019; Zhao et al., 2023).

**TABLE 1:** Classifications and representative diseases of IMIDs.

Affected organ or tissue	Representative diseases
Joint and bone diseases	Rheumatoid arthritis, psoriatic arthritis, axial spondyloarthritis, Juvenile idiopathic arthritis, ankylosing spondylitis
Skin disorders	Psoriasis, atopic dermatitis, Alopecia areata, Vitiligo, Hidradenitis suppurativa, Behcet disease
Gastrointestinal diseases	Crohn’s disease, ulcerative colitis
Central and peripheral nervous system conditions	Multiple sclerosis, Myasthenia gravis
Eyes disease	Uveitis, Neuromyelitis optica spectrum disorders
Pulmonary disease	Asthma
Blood vessel disorder	ANCA-associated vasculitis, Giant cell arteritis
Other multi-system overlap types	Systemic lupus erythematosus, IgG4-related disease, Sjögren’s syndrome, Sarcoidosis

The treatment landscape for IMIDs has undergone several evolutionary phases. At the close of the 20th century, systemic glucocorticoids were the mainstay of therapy. While these medications delivered impressive results initially, their efficacy tended to wane over time, accompanied by a buildup of toxic side effects. Subsequently, conventional synthetic disease-modifying anti-rheumatic drugs (DMARDs) like methotrexate and azathioprine, were introduced and have continued to be first-line recommendations in clinical guidelines for conditions such as rheumatoid arthritis (RA), systemic lupus erythematosus (SLE), psoriatic arthritis (PsA), and other related disorders. They offer a great advantage of simultaneously targeting multiple inflammatory pathways, with early and continuous use markedly lowering disability risk (McInnes and Gravallese, 2021; Williams and Meyers, 2002). However, these DMARDs are hindered by major drawbacks including delayed onset, non-specific immunosuppression, increasing infection, myelosuppression and potential hepatorenal toxicity (Muñoz-Martínez et al., 2021). Recently, the recognition of IMIDs sharing immunopathogenic pathways has spurred a therapeutic shift from biologics to next-generation small-molecule drugs that precisely intervene critical cytokines or signaling pathways; e.g., biologics directed against TNF and IL-17A/IL-23, as well as small-molecule JAK inhibitors, have been widely adopted in clinical practice for the treatment of IMIDs. It should be noted, however, that small-molecule TNF inhibitors have yet to reach this milestone and remain in the early phases of clinical testing. But as research progresses, the limitations of both biologics and small-molecule drugs have become increasingly evident. For instance, JAK inhibitors can raise the risk of herpes zoster, while TNF inhibitors may reactivate latent tuberculosis, underscoring the persistent therapeutic challenges in managing IMIDs (Sunzini et al., 2020; Alıravcı et al., 2025). Given the potential of plant-derived compounds as a source of new therapeutic candidates, natural extracts have attracted increasing attention in the search for additional treatment options for IMIDs in recent years. Proanthocyanidins (PCs), a prominent branch of the plant-polyphenol family, abound in fruit peels, seeds, flowers, and nuts; their remarkably diverse structures confer potent antioxidant, anti-inflammatory and immunomodulatory properties (Nandakumar et al., 2008). With a wide safety range and excellent tolerability, PCs are now regarded as ideal adjuvant or preventive drugs for IMIDs. Here, we dissect the pathogenesis of these diseases and review the mechanisms of action of PCs in the most common IMIDs, outlining the therapeutic mechanisms that should guide their wider use in more IMIDs.

## 2 Etiology and pathogenesis of IMIDs

The introduction of IMIDs concept marks a shift in the medical understanding of them, transitioning from organ-based symptomatic relief to mechanism-based treatment (McInnes and Gravallese, 2021). Many clinically unrelated diseases, in fact, share similar immune dysregulation mechanisms in IMIDs. Although their precise pathogenesis remains elusive, growing evidence about etiology and pathogenesis links the onset and progression of these disorders to genetic susceptibility, environmental factors, microbiota dysbiosis, and dysregulated cytokine networks and signaling cascades.

### 2.1 Etiology of IMIDs

#### 2.1.1 Genetic susceptibility

A large number of epidemiological and genomic studies have confirmed the genetic basis of IMIDs. For instance, in inflammatory bowel disease (IBD), the risk of disease among relatives is significantly higher than that in the general population, and the incidence rate is higher in monozygotic twins than in dizygotic twins (David et al., 2018). Meanwhile, early studies had already shown that nucleotide-binding oligomerization domain-containing protein 2 (NOD2) gene mutations remarkably enhance crohn’s disease (CD) risk (Hugot et al., 2001). Collectively, these data implicate a potent genetic contribution. Human leukocyte antigen (HLA) gene variation is the most common genetic risk factor for IMIDs, with the HLA-DRB1*04 gene variant being common in patients with RA, and psoriasis is associated with the HLA-C*06:02 gene abnormality (Winchester et al., 2012; Gregersen et al., 1987). The close relationship between ankylosing spondylitis and HLA-B27 is also well-known (Brown et al., 2020). HLA variation affects antigen presentation capacity, thereby altering the T cell recognition threshold for self-antigens, inducing immune tolerance imbalance, and consequently leading to the development of IMIDs (Ishigaki et al., 2022; Dendrou et al., 2018).

#### 2.1.2 Environmental factors

Genetic susceptibility alone, however, does not determine the onset of disease. Instead, it interacts with environmental factors to contribute to IMIDs occurrence. Smoking, a major environmental risk factor, has garnered academic interest due to its involvement in the initiation and progression of various IMIDs. Current research implicates tobacco smoke in the pathogenesis of IMIDs via multiple pathways. For instance, Chen et al. reported that smoking appears to influence disease localization in CD, with smokers more likely to develop ileitis than colitis—a finding potentially attributable to the preferential accumulation of nicotine in the ileum (Chen et al., 2022). Moreover, smoking interacts with genetic susceptibility; studies show that the combination of smoking and NOD2 variants markedly increases the risk of disease (Helbig et al., 2012). It is also observed that tobacco use attenuates therapeutic responses to biologic agents and small-molecule inhibitors in patients with IMIDs. Thus, smoking cessation should be regarded as an essential adjunct in the management of IMIDs, capable of improving disease prognosis and potentiating the efficacy of targeted therapies (Nicolaides et al., 2021). Beyond tobacco, poor foods rich in additives damage the intestinal mucus barrier, facilitating bacterial translocation and inducing colitis (Bancil et al., 2021; Laudisi et al., 2019). Similarly, obesity is associated with elevated incidence of IBD, RA and PsA, while heavy alcohol intake predisposes to both RA and psoriasis (López-Ferrer et al., 2022; Chan et al., 2022; Cush, 2022; Higgins, 2000).

#### 2.1.3 Microbiota dysbiosis

Existing evidence supports a correlation between IMIDs and gut microbiota. Typically, patients with IBD exhibit a general reduction in gut microbial diversity, a decline that undermines the stability of the intestinal ecosystem and leaves it more vulnerable to external environmental disturbances (Round and Mazmanian, 2009; Xavier and Podolsky, 2007). Concurrently, the increased abundance of bacterial phyla with pro-inflammatory potential, like Proteobacteria, has also been reported in the pathogenesis of various IMIDs, while fluctuations in the abundance of specific gut bacteria appear to track the clinical stage of these disorders. For example, a decrease in the anti-inflammatory bacterium *Faecalibacterium prausnitzii* indicates the initial stage of postoperative recurrence in CD patients, whereas *Prevotella copri* is more common in early-stage RA (Diaz et al., 2024; Sokol et al., 2008; Scher et al., 2013). Sometimes, infections—bacterial, viral, or fungal—also trigger IMIDs. Psoriasis is a typical case: removal of tonsils often ameliorates skin scores of psoriasis, possibly by eliminating a reservoir of β-haemolytic streptococci that can activate tonsillar T cells and direct them to the skin, amplifying psoriatic lesions (Gudjonsson et al., 2003; Valdimarsson et al., 2009; Thorleifsdottir et al., 2017). Periodontitis, similarly, raises RA risk—an association increasingly traced to by *Porphyromonas gingivalis* and *Aggregatibacter actinomycetemcomitans* (Gómez-Bañuelos et al., 2019; Ungprasert et al., 2017). Additionally, Epstein-Barr virus (EBV) may induce the onset of IMIDs by driving chronic inflammation and a lasting cytokine imbalance (Tan and Smolen, 2016).

### 2.2 Pathogenesis of IMIDs

#### 2.2.1 Immune cell dysregulation

##### 2.2.1.1 Th17/Treg cell imbalance

Th17 cells and Treg cells are two main subgroups of CD4^+^ T cells, and their balance is of great significance for maintaining immune homeostasis. Studies have found that IMIDs patients often have a coexisting state of Th17 cell hyperactivation and Treg cell functional suppression or quantity reduction, this immune imbalance that promotes the occurrence and development of IMIDs (Park MK. et al., 2011; Hartwig et al., 2018). Under physiological conditions, Th17 cells police the gut barrier, strengthening mucosal immunity against microbes invasion; in IMIDs, however, they slip into overactivation and dysfunction and their secreted cytokines—IL-17A, IL-17F, interleukin-21 (IL-21) and interleukin-22 (IL-22)— become the principal accelerants of the inflammatory cascade (Raza et al., 2012; Dong, 2008). Within the evolving inflammatory microenvironment, Th17-derived cytokines push macrophages to release interleukin-1β (IL-1β), interleukin-6 (IL-6), and TNF-α, escalating the inflammatory response, while IL-17A/F concurrently recruits neutrophils to the site of inflammation. Neutrophils then exacerbate tissue damage through the discharge of hydrolases and reactive oxygen species, thereby amplifying inflammation and establishing a self-amplifying positive-feedback loop that underpins the pathogenesis of IMIDs (Bunte and Beikler, 2019; Chen et al., 2019; Lopetuso et al., 2025; Shin et al., 2017). Treg cells, by contrast, enforce peripheral tolerance: they suppress effector T-cell expansion and function via IL-10- and TGF-β-mediated signaling coupled with contact-dependent inhibitory pathways, thereby safeguarding immune homeostasis (Hartwig et al., 2018). In various IMIDs, Treg deficits—whether numeric, functional, or destabilized by the local milieu—are key contributor to diseases progression. In IBD, for instance, the mucosa shows a reciprocal shift: Th17 cells expand while Treg numbers collapse, locking the gut into chronic inflammation (Hu et al., 2025). In RA, conversely, the synovial fluid in joint cavity is paradoxically flushed with Treg cells, yet their suppressive ability is blunted; they fail to curb T-cell proliferation or cytokine release, leaving pro-inflammatory mediators out of control and allowing synovitis or cartilage erosion to become chronic (Yan et al., 2022). This Th17/Treg imbalance—a systemic tilt toward hyperactive pro-inflammatory signaling and fading anti-inflammatory responses—drives organ-targeted inflammatory injury and marks a critical gateway to IMID development.

##### 2.2.1.2 Th1/Th2 cell imbalance

Dysregulation of helper T (Th) cell subsets, especially the Th1/Th2 imbalance, is a well-established contributor to IMID pathogenesis. Th1 cells mainly secrete cytokines Interferon-γ (IFN-γ), TNF-β and IL-2, driving cell-mediated immunity; Th2 cells, by contrast, release IL-4, IL-5, IL-10 and IL-13, coordinating humoral immunity and allergic reactions (Kuek et al., 2007). A Th1/Th2 imbalance is implicated in a broad spectrum of IMIDs, wherein overactivation of one subset or failure of bidirectional cross-regulation triggers loss of self-tolerance and subsequent autoreactivity. Th1-dominant IMIDs, such as CD and RA, often exhibit a predominance of Th1-type immune responses; in these diseases, high-level Th1 cytokines (e.g., IFN-γ and TNF-α) appears, further facilitating macrophage activation and cytotoxic T cell responses, ultimately exacerbating tissue damage (Yan et al., 2022; Luo et al., 2022). In Th2-dominant IMIDs—typified by allergic asthma, atopic dermatitis (AD) and certain autoimmune diseases, instead, the immune response is skewed toward a Th2 profile with cytokines hallmarked by IL-4, IL-5, and IL-13 (Stark et al., 2019); these cytokines encourage B cells-secreted high-level immunoglobulin E (IgE) production and eosinophil activation, thereby orchestrating the transition to chronic inflammation (Ogulur et al., 2025).

#### 2.2.2 Abnormalities of the key cytokines and signal pathways

##### 2.2.2.1 TNF-α in IMIDs

TNF-α is a multifunctional cytokine secreted by macrophages, T cells, natural killer cell, and other immune cells (Smookler et al., 2006). It primarily binds to two receptors: tumor necrosis factor receptor 1 (TNFR1), which widely expresses in all cell types and TNFR2, whose presence is more exclusive to immune cells and endothelial cells (Yang et al., 2018). TNF-α, through receptor-mediated downstream signaling pathways, activates the nuclear factor-κB (NF-κB) and mitogen-activated protein (MAPK) signaling pathways, leading to the release of multiple pro-inflammatory cytokines, thereby causing damage to the body’s tissues and organs. Upon sensing PAMPs (pathogen-associated molecular patterns), DAMPs (damage-associated molecular patterns) or pro-inflammatory cytokines, cells assemble the IκB kinase (IKK) complex that marks Inhibitor of kappa B (IκB) for phosphorylation-ubiquitination–proteasomal destruction. The IκB-degradation-released NF-κB dimers then translocate to the nucleus and initiate a transcriptional burst that puts TNF-α at the top of an amplified inflammatory cascade. Secreted TNF-α feeds back through TNFR1/2 to sustain IKK–NF-κB signaling, while simultaneously inducing cytokines (IL-6, IL-1β, IL-17), chemokines, adhesion molecules, and matrix metalloproteinases (MMPs), an auto-escalating loop that converts a transient stimulus into persistent inflammatory response (Adel et al., 2019; Navarro-Compán et al., 2023).

Among multiple IMIDs, excessive or persistently high TNF-α acts as a principal driver of disease progression. Binding of TNF-α to cell-surface TNFR triggers NF-κB activation; in turn, NF-κB translocates to the nucleus and boosts further TNF-α transcription, forming a positive inflammatory feedback that fuels both the initiation and development of IMIDs. In psoriasis, for instance, this cytokine not only stimulates epidermal keratinocytes hyperproliferation but also intensifies downstream inflammatory signaling cascades; Together with IL-17 and IL-23, it markedly exacerbates psoriatic lesions (Menter et al., 2021). In RA and PsA, TNF-α serves as a potent stimulator of osteoclasts, inducing stromal cells to secrete collagen and matrix-degrading enzymes, which consequently leads to IMID-associated tissue damage (Park et al., 2021; Yuan et al., 2025). Among IBD patients, TNF-α ignites intestinal mucosal inflammation and ulceration, recruits inflammatory cell and sustains a chronic inflammatory state (De Santis et al., 2017). Apart from that, elevated TNF-α remarkably induces Treg cells dysfunction, thereby eroding their immunosuppressive capacity and driving IMIDs forward (Nie et al., 2013).

##### 2.2.2.2 IL-23/IL-17 axis in IMIDs

IL-23 is a cytokine secreted predominantly by antigen-presenting cells, notably macrophages and dendritic cells (Xio et al., 2022). It drives the induction, proliferation, differentiation and survival of Th17 cells, which in turn secrete a series of effector cytokines headed by IL-17. By binding to its specific receptor IL-23R, IL-23 excites the JAK-STAT pathway, steering CD4^+^ T cells toward Th17 lineage, promoting their massive proliferation and IL-17-related factors release, and inducing downstream pro-inflammatory cascades (García-Domínguez, 2025; McGeachy et al., 2009). Although multiple cell types can produce IL-17, Th17 cells represent the primary source, particularly under the drive of IL-23. Meanwhile, as a pro-inflammatory cytokine, the effects of IL-17 extend beyond Th17 cells. Once secreted by Th17 cells, IL-17 can act on various target cells, eliciting diverse biological effects. The IL-23/IL-17 axis operates through IL-23-induced differentiation and proliferation of Th17 cells, which subsequently secrete IL-17 (McAleer and Kolls, 2011; Schinocca et al., 2021). IL-17 then acts on multiple target cells, triggering extensive inflammatory cascades, including the production of pro-inflammatory mediators and chemokines, aberrant cell proliferation, as well as tissue damage and remodeling, ultimately contributing to the pathogenesis and progression of IMIDs. Existing evidence supports the IL-23/JAK1/STAT3 axis within psoriatic lesions as a critical determinant of Th17 cells and T-cell subset dynamics. Here, IL-23 attaches onto its receptor and phosphorylates JAK1 and STAT3, thereby stimulating the proliferation and differentiation of Th17 cells. The resulting surge of IL-17 works on keratinocytes to encourage their abnormal proliferation and to release inflammatory mediators, propelling the epidermal hyperplasia and sustained cutaneous inflammation that drive psoriasis forward (Guo et al., 2023). Beyond that, IL-17 promote the proliferation of synovial fibroblast-like cells and the secretion of inflammatory MMPs via the JAK-STAT signaling. In concert with TNF-α, it amplifies the inflammatory response, culminating in progressive damage to both connective tissue and bone (Lee et al., 2014). As shown in Figure 1.

**FIGURE 1:**
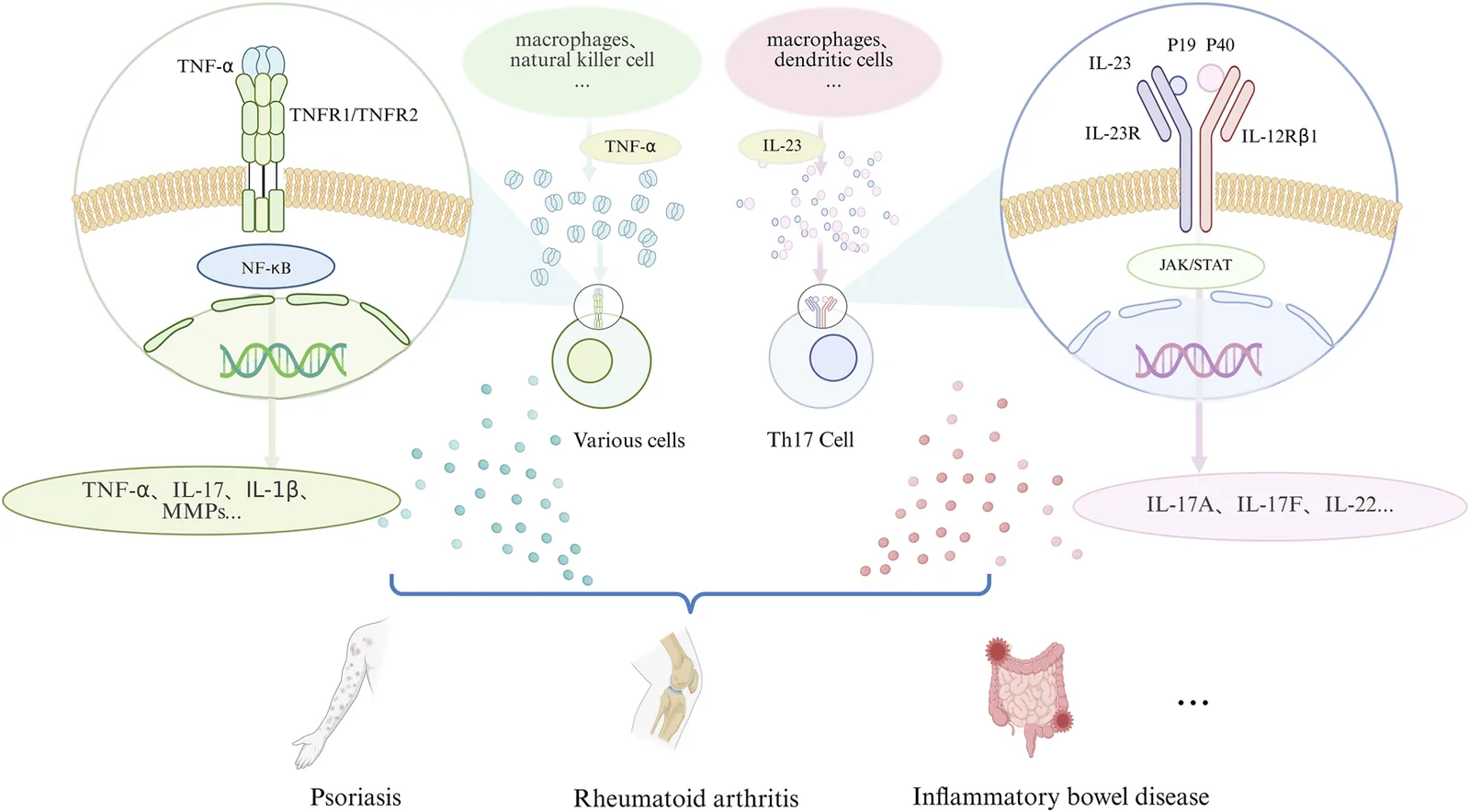
Pathogenic mechanisms of TNF-α and IL-23/IL-17 axis in IMIDs. TNF-α, secreted by diverse immune cells, binds to cell-surface TNFR1/2 to activate the NF-κB pathway, thereby inducing the production of TNF-α itself along with cytokines (IL-1β, IL-17), chemokines, adhesion molecules, and MMPs. IL-23, produced by antigen-presenting cells, engages the IL-23 receptor to activate JAK-STAT signaling, driving Th17 cell differentiation, proliferation, and the secretion of IL-17-dominated pro-inflammatory cytokines. The pro-inflammatory mediators generated through these processes act upon the skin, joints, and gastrointestinal tract, resulting in the development of classic IMIDs, including psoriasis, RA, and IBD, etc*.*

Alongside the aforementioned mediators, cytotoxic T-lymphocyte antigen 4 (CTLA-4) has attracted considerable interest in the context of IMIDs such as RA, PsA and psoriasis. By negatively regulating T-cell activation and restraining auto-reaction, it serves as a critical brake on disease-driving immunity (Cutolo et al., 2016). Meanwhile, phosphodiesterase-4 (PDE-4) inhibitors—exemplified by apremilast—are now recognized players in PsA and psoriasis. By selectively blocking PDE-4, they raise intracellular cyclic adenosine monophosphate (cAMP), dampen pro-inflammatory cytokines release and halt the downstream inflammatory cascade (McInnes and Gravallese, 2021; Picchianti-Diam et al., 2021).

## 3 Characteristics of PCs

### 3.1 Distribution, structure and classifications of PCs

PCs, firstly isolated from the red testae of peanut kernels, were demonstrated the ability of restoring capillary integrity, soon followed by the recovery of analogous polyphenols from hawthorn berries and grape seeds, coining the name “proanthocyanidins” (Weseler and Bast, 2017). PCs are a widely distributed secondary metabolite of polyphenols in the plant kingdom and are one of the most consumed flavonoids in Western diets (Aron and Kennedy, 2008). Grape seeds and grape skins are among the highest sources in current research. Pine bark is another important source. There is now a commercial product, Pycnogenol®, derived from the bark extract of the French maritime pine (*Pinus pinaster*), with its proanthocyanidin content reaching up to 85% (Szwajkowska-Michałek et al., 2020). In apples, PCs are the most predominant phenolic component, accounting for over 80% of the total polyphenols, and they are mostly in a polymerized form (Wojdyło et al., 2008). From a structural perspective, PCs are oligomeric or polymeric chains of flavan-3-ols linked by carbon-carbon (C-C) bonds, and belong to the large flavonoid family (Ma and Zhang, 2017). Their basic building blocks include catechin, epicatechin, gallocatechin, epigallocatechin, afzelechin, and epiafzelechin. Notably, five structural features determine the diversity of PCs: the flavan-3-ol monomers, the interflavan linkage modes, the degree of polymerization (DP), the stereochemistry, and the hydroxylation pattern. By DP, it treats dimers to tetramers as oligomers, while pentamers and beyond are ranked as polymers (Lim and Ha, 2021; Das et al., 2023). In addition, according to the linkage pattern, PCs can be divided into two types: B-type PCs, which are formed through the common C4→C8 or C4→C6 interflavan linkages, and the rarer A-type PCs, which contain an additional C2→O7 ether bond between flavan-3-ol units (Diaz et al., 2024; Rodríguez-Pérez et al., 2019). The structural profiles of PCs vary considerably across different sources. B-type PCs are predominantly found in grape seeds, whereas cranberries are known for their distinctive A-type linkages, and cinnamon also contains predominantly A-type PCs, which are its most abundant phenolic constituents, accounting for about 23.2% of the crude extract (Tanabe et al., 2011; Mateos-Martín et al., 2012). Litchi pericarp is likewise rich in PCs, which can constitute approximately 60% of the extracted polyphenols, mainly in the form of oligomers with an average degree of polymerization around 4 (Miranda-Hernández et al., 2019). In addition to the aforementioned sources, cocoa, berries, nuts, and leguminous forages are also important sources of PCs, and Chinese jujube has also been developed as a novel source of PCs (Jonker and Yu, 2017; Lu et al., 2025). As shown in Table 2.

**TABLE 2:** Basic structures and representative plants of type A and B proanthocyanidins.

Representative plant	Basic structure	
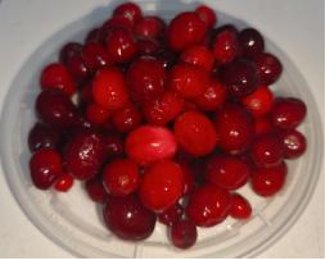 Cranberry	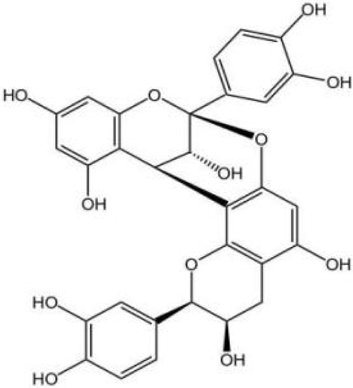 Proanthocyanidin A1	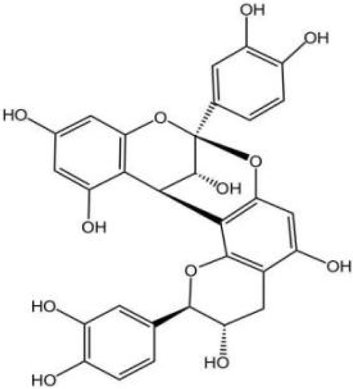 Proanthocyanidin A2
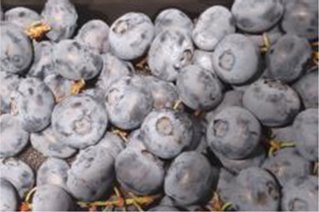 Blueberry	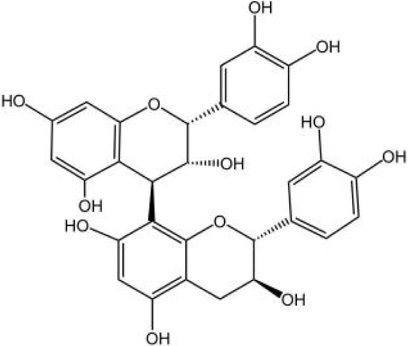 Proanthocyanidin B1	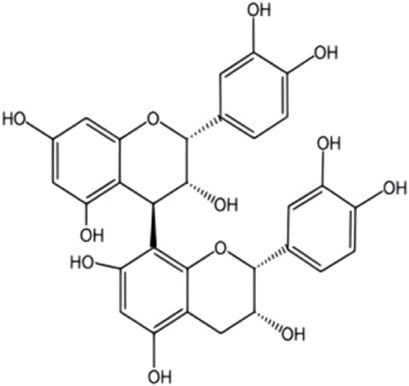 Proanthocyanidin B2
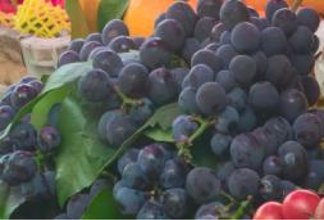 Grape	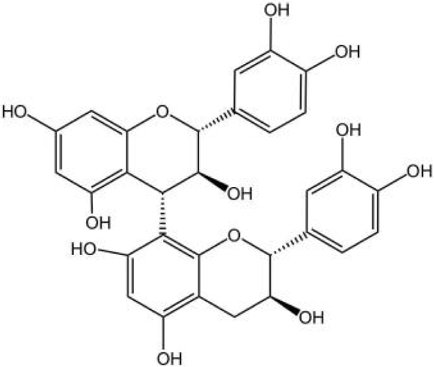 Proanthocyanidin B3	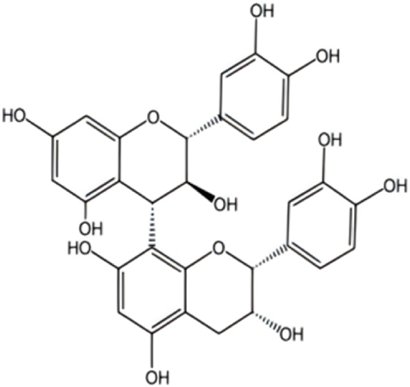 Proanthocyanidin B4
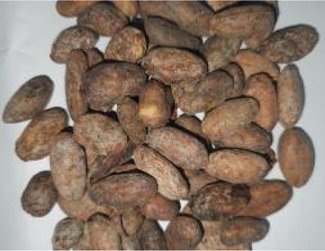 Cocoa beans	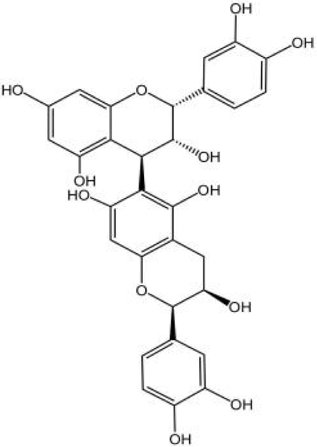 Proanthocyanidin B5	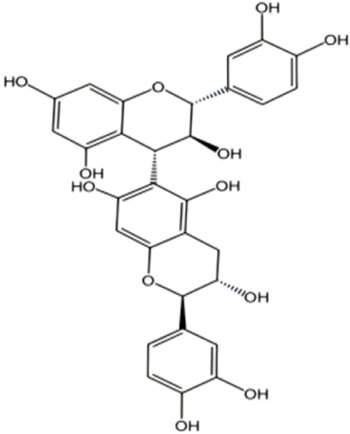 Proanthocyanidin B6
​	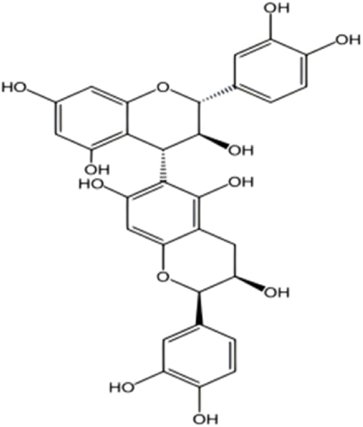 Proanthocyanidin B7	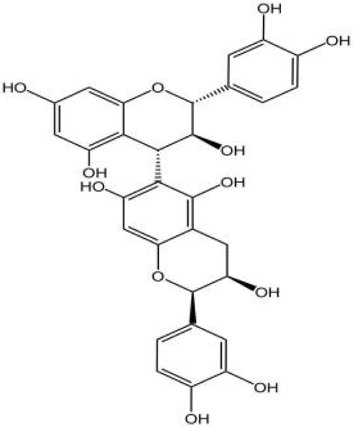 Proanthocyanidin B8

### 3.2 Absorption and metabolism of PCs

PCs rank among the most abundant yet least absorbed dietary polyphenols, due to their size and structural complexity (Bovee et al., 2015). Upon oral ingestion and mastication, PCs traverse the esophagus and enter the stomach. The majority of PCs remain relatively stable in the acidic gastric environment. Only a minor fraction may undergo structural alterations, such as depolymerization or polymerization, due to gastric acid exposure; however, these changes are typically negligible, and most PCs proceed into the small intestine in their original or near-original forms (Rios et al., 2002; Mannino et al., 2021; Zhang et al., 2025). The absorbed flavan-3-ol monomers and oligomers undergo Phase II metabolism in the intestinal wall and liver, where they are conjugated through glucuronidation, sulfation, and O-methylation, catalyzed respectively by uridine diphosphate glucuronosyltransferase (UDPGT), sulfotransferase (SULT), and catechol-O-methyltransferase (COMT). The resulting conjugated metabolites then enter the systemic circulation. These Phase II conjugation reactions not only alter the chemical form of PCs, but also directly affect their *in vivo* distribution and final routes of excretion; some conjugates can be re-secreted into the intestine via the bile or cleared by the kidneys and excreted in the urine (Zeng et al., 2020).

While monomeric flavan-3-ols are readily absorbed in the small intestine, larger oligomers and polymers transit intact to the colon, where studies have shown that PCs are progressively degraded by the gut microbiota to generate core metabolites represented by hydroxyphenyl-γ-valerolactones and phenylvaleric acids (Mena et al., 2019). These microbial metabolites, not the original high-molecular-weight compounds, are the chief mediators of PC-linked health benefits (Monagas et al., 2010). With DP increase, PCs absorption significantly goes down; the pentamer reaches the upper limit of absorption, whereas those at DP > 4 barely cross epithelia (Choy et al., 2013). A-type PCs possess slightly higher bioavailability than B-type owing to additional ether bond between C2 and O-C7, while co-ingestion of carbohydrates, proteins and fiber may modulate the absorption of PCs (Bohn, 2014; Molinar-Toribio et al., 2017; Appeldoorn et al., 2009). Individual variation further complicates the absorption and metabolism of PCs. On the one hand, the composition of the gut microbiota differs greatly among individuals, so that different individuals convert PCs into active metabolites with different types and efficiencies (Mena et al., 2019). On the other hand, host genetic polymorphisms of metabolic enzymes are also involved; for example, COMT genotype differences can affect the rate of O-methylation and the absorption and metabolism of catechin-type compounds, further amplifying the differences in the response of the same PCs intervention among individuals (Miller et al., 2012). To enhance the PCs bioavailability, extensive efforts have been made to explore nanotechnology as a crucial strategy for overcoming bioavailability barriers. Nanoformulations can protect PCs from premature degradation, enable targeted delivery and facilitate sustained release at lower doses thereby enabling the efficient utilization of PCs (Yang et al., 2020; Tian et al., 2020). As shown in Figure 2.

**FIGURE 2:**
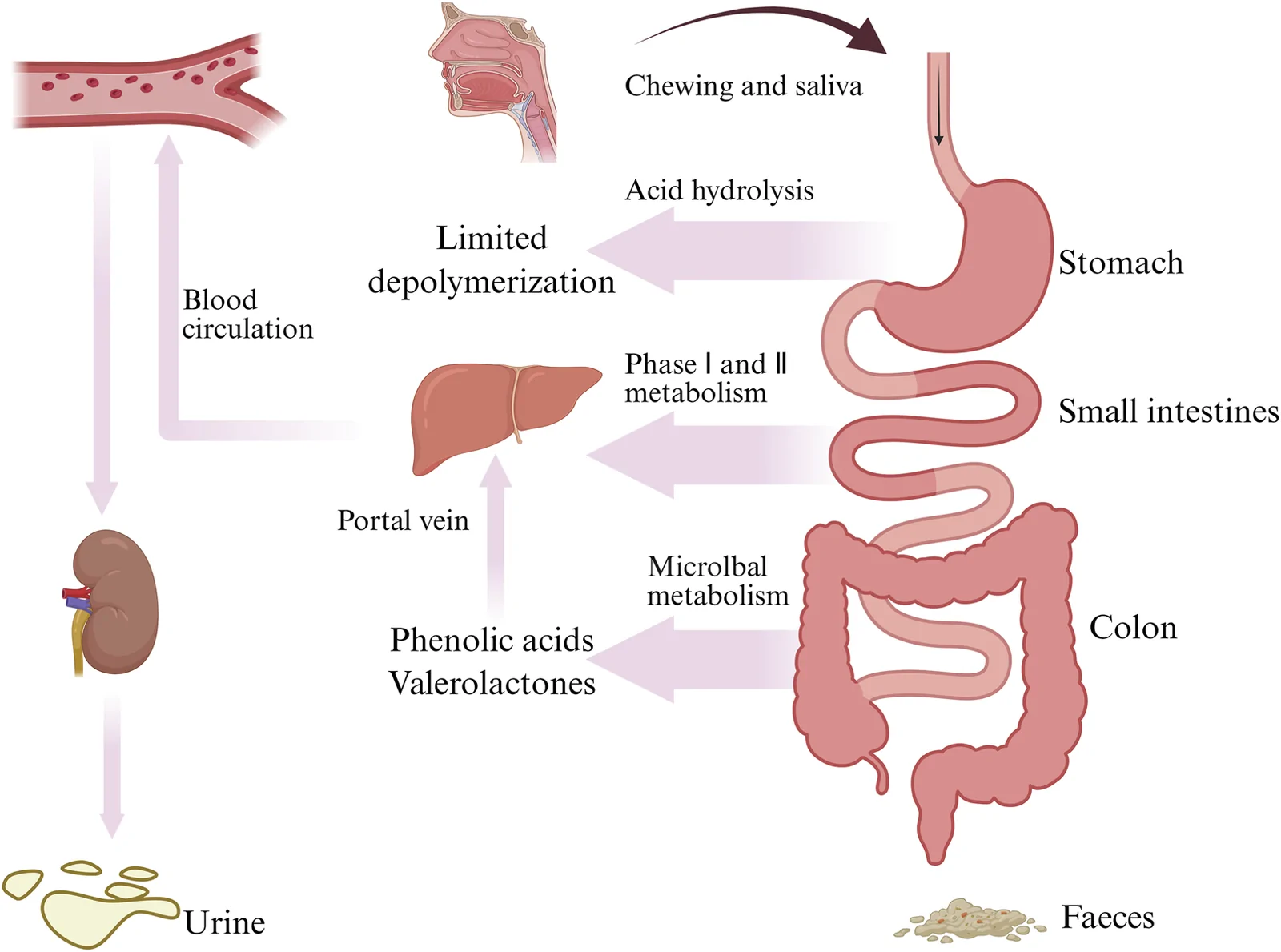
The absorption, metabolism, distribution, and excretion of PCs. After oral administration, PCs are first entering the stomach, where they undergo limited degradation in acidic conditions. Most PCs are subsequently absorbed and metabolized in the small intestine, then transported to the liver for Phase I and Phase II biotransformation. The unabsorbed residual fraction is fermented by the colonic microbiota, yielding a diverse array of metabolites. These metabolites then enter systemic circulation and distribute to various organs, where they exert their pharmacological effects, ultimately being excreted through urine and faeces.

### 3.3 Biological activities of PCs

NF-κB is a central transcription factor controlling the expression of numerous pro-inflammatory cytokines, including TNF-α, IL-1β, and IL-6, and is therefore a pivotal hub of inflammatory gene regulation. Anti-inflammatory research has confirmed, PCs exert a direct and broad inhibitory effect on this pathway. In lipopolysaccharide (LPS)-stimulated RAW264.7 macrophages, GSPE suppress NF-κB signaling and thereby downregulate inducible nitric oxide synthase (iNOS) at the transcriptional level, reducing the production of nitric oxide (NO) and prostaglandin E2 (PGE2) (Terra et al., 2007). Because NF-κB occupies a position upstream of multiple pro-inflammatory cascades, its inhibition by PCs simultaneously dampens the expression of several downstream inflammatory genes, representing one of the most fundamental molecular bases of their anti-inflammatory activity. The mitogen-activated protein kinase (MAPK) family, comprising extracellular signal-regulated kinase (ERK), c-Jun N-terminal kinase (JNK), and p38, also transduces inflammatory signals. PCs can exert anti-inflammatory effects by inhibiting MAPK signaling. GSPE act on both the MAPK and NF-κB pathways when suppressing LPS-induced inflammatory mediator expression (Chu et al., 2016). OS and inflammation are intimately intertwined and mutually reinforcing, and nuclear factor erythroid 2-related factor 2 (Nrf2) is a key transcription factor governing cellular antioxidant defense. PCs can activate the Nrf2/antioxidant response element (ARE) signaling pathway. It has been demonstrated that PCs stabilize and activate the Nrf2/ARE antioxidant response by inhibiting the ubiquitinated degradation of Nrf2 (Shuhua et al., 2022). In terms of immune regulation, PCs also exert immunomodulatory effects by regulating the balance of T cell subsets in adaptive immunity. In a collagen-induced arthritis model, GSPE differentially regulated the proportions of Tregs and pathogenic T cells, promoting Tregs while suppressing Th17 cells, thereby inducing a Th17/Treg rebalance and alleviating arthritis (Ahmad et al., 2013). In addition to the signaling pathways and T cell subsets discussed above, PCs also act on innate effector cells. Procyanidin C1 from apple inhibits FcεRI-mediated mast cell activation and degranulation, while oligomeric PCs inhibit the maturation of dendritic cells, thereby reducing their antigen-presenting capacity (Nakano et al., 2008; Li et al., 2017).

Meanwhile, PCs show a potential anticancer promise by inducing tumor-cell apoptosis and halting cell migration or invasion (Yu A. et al., 2025; Li et al., 2025). PCs also curb metabolic diseases and infections by modulating the gut microbiota and exert immunomodulatory effects (van Duynhoven et al., 2011). In addition to their antibacterial effects, in which cranberry-derived PCs block periodontal pathogens, inhibit MMP-2/9, and disrupt biofilms, they can also specifically inhibit the adhesion of uropathogenic *Escherichia coli* P-fimbriae to urothelial cells, thereby preventing urinary tract infections (Nie et al., 2023; Hah et al., 2017; Howell et al., 2005). PCs not only work alone but also boost other therapies. In a mouse psoriasis model, topical PCs plus IL-17A antibody shaved 35% off the PASI score and thinned the epidermis by 20%, showing that PCs can push drugs through skin and elevate biologics efficacy (He et al., 2025).

## 4 Therapeutic role of PCs in common IMIDs

IMIDs are characterized by chronic inflammation and abnormal immune responses that strike almost any organ. Fortunately, PCs—as safely natural plant polyphenols with strong anti-inflammatory, antioxidant and immunomodulatory properties—dampen these attacks by blocking JAK/STAT and NF-κB signaling, rebalancing T lymphocyte subsets, and neutralizing ROS to arrest oxidative damage. The following parts detail how PCs exert these effects in the most common IMIDs, like psoriasis, IBD, AD, RA and asthma.

### 4.1 PCs in psoriasis

Psoriasis is an immune-mediated chronic skin disease driven by genetics, environment, oxidative stress (OS) and immune dysregulation (García-Pérez et al., 2010; Yang et al., 2022). The core of its pathogenesis lies in the abnormal interaction between the innate immune cells of the skin (such as dendritic cells) and the adaptive immune cells (especially Th17 cells). Among them, IL-23 produced by dendritic cells promotes the differentiation of Th17 cells and the secretion of pro - inflammatory cytokines such as IL-17 and IL-21. Subsequently, IL-17 drives the excessive proliferation, abnormal differentiation of keratinocytes and the release of chemokines, forming a positive “immune - epithelial” feedback loop, which constitutes the key pathological link of the disease (Griffiths et al., 2021). Furthermore, the role of OS in the onset of psoriasis is receiving increasing attention, OS-induced ROS accumulation stimulates pro-inflammatory cytokines and VEGF secretion, promoting angiogenesis and activating inflammatory signaling pathways that produce characteristic pathological features. Meanwhile, the Th17/Treg balance shifts toward hyperactive Th17 cells and suppressed Treg function (Lai et al., 2018). Besides, cutaneous dysbiosis—enriched *Staphylococcus epidermidis*, *Streptococcus*, and pro-inflammatory bacterial phyla (Actinobacteria, Firmicutes, Proteobacteria)—exacerbates and sustains the disease process (Burlec et al., 2024).

In established psoriasis-like cell models, PCs not only strengthened antioxidant defenses (superoxide dismutase, catalase, glutathione) along with ROS and malondialdehyde (MDA) reduction, but also inhibited the phosphorylation of JAK2, STAT3 and AKT, thereby blocking inflammatory signaling (Zhao et al., 2022). In addition, PCs tilt the cytokine profile from pro-inflammatory (TNF-α, IL-17) toward anti-inflammatory (IL-10), collectively restoring skin immune homeostasis (Toda et al., 2020). And in Zhao’s model of psoriasis-like keratinocytes (HaCaT), PCs combat psoriasis through nuclear factor erythroid 2-related factor 2 (Nrf2)-dependent induction of heme oxygenase-1 (HO-1) to quench ROS and shield keratinocyte DNA (Zhao et al., 2022). Meanwhile, in the psoriasis-like mouse model induced by imiquimod (IMQ), it was confirmed that PCs could significantly improve the psoriasis-like skin symptoms, inhibit inflammatory responses, and alleviate OS damage, they rebalance immunity via IL-23/IL-17 blockade that silences Th17-derived inflammatory factors (IL-17A/F, IL-21 and IL-22) and equilibrates Treg/Th17 ratio (Yu L. et al., 2025; Zhong et al., 2022). Meanwhile, PCs brake STAT3-driven keratinocyte hyperproliferation to thin the epidermis and normalize cornification—converging antioxidant, immunomodulatory, anti-proliferative and anti-angiogenic activities into overall relief of erythema, plaque and scaling (Yang et al., 2022).

### 4.2 PCs in IBD

IBD, primarily CD and ulcerative colitis (UC), denotes a group of chronic conditions characterized by mucosal inflammation, barrier disruption and microbiota dysbiosis that produce abdominal pain, severe diarrhea, fatigue and weight loss. Its undesirables, containing high prevalence, refractory course, and erosion of life quality, have made IBD a major global health threat (Liu et al., 2025; Zhang and Li, 2014). Mounting evidence now pinpoints OS, aberrant immune responses, and microbiota imbalance as central drivers of IBD, yet current therapies still rely on colonic immunosuppression that invites drug toxicity and immune damage (Yang et al., 2025).

PCs, with diverse bioactivities, protect intestinal epithelial cells and scavenge ROS, making them promising drug candidates for IBD (Diaz et al., 2024). Mechanistically, they prevent the inflammatory process in the colon and tilt the cytokine balance by down-regulating pro-inflammatory factors TNF-α, IL-1β, IL-6, IL-12 and IFN-γ, while elevating anti-inflammatory cytokines IL-4 and IL-10. This dual effect damps the colonic inflammatory cascade (TNF-α, IL-1β and IL-6-dominant inflammation) (Cai et al., 2019), curbs leukocyte recruitment and preserves epithelial integrity, collectively alleviating mucosal damage in UC. In addition, macrophages drive IBD through releasing extensive pro-inflammatory cytokines, chemokines and ROS; conversely, this condition could be held back by PCs that reduce macrophage-produced ROS further to suppress MMP9, block NF-κB signaling and dismantle NOD-like receptor protein 3 (NLRP3) inflammasome in murine colitis (Han et al., 2019; Sahoo et al., 2023; Shi et al., 2023). Recent work shows that a balanced gut microbiota safeguards colonic physiology and modulates immunity; probiotics defend against inflammation, whereas pathogen expansion in the colon enhances IBD risk (Sahoo et al., 2023). However, using the dextran sulfate sodium (DSS)-induced mouse model of colitis, we found that PCs, nevertheless, positively maintain intestinal homeostasis and alleviate IBD by enriching beneficial bacteria (e.g., *Lactobacillus* and *Bifidobacterium*) while suppressing harmful species such as *Sutterella* and *Bacteroides* (Cai et al., 2019). They, moreover, restore intestinal barrier function through enhancing epithelial tight junction integrity, further to work well in IBD (González-Quilen et al., 2020). Notably, Bitzer et al. demonstrated that PCs with DP ≥ 7 exhibit superior barrier-protective activity, suggesting that their polymerization degree critically influences this function (Bitzer et al., 2015).

Beyond that, iron-dependent ferroptosis, characterized by lipid peroxidation and ROS accumulation, drives intestinal epithelial cell death and chronic inflammation in UC. Consequently, modulating ferroptosis offers a promising therapeutic avenue for IBD. Wang et al. demonstrated that PCs originating from cranberries attenuated DSS-induced IBD mouse model through regulate ferroptosis-related gene expression, upregulating glutathione peroxidase 4 (GPX4), solute carrier family seven member 11 (SLC7A11), HO-1, and ferritin light chain to enhance colonic antioxidant defenses and mitigate oxidative damage, highlighting their significance in colon health and IBD pathophysiology (Wang et al., 2024; Long et al., 2024).

### 4.3 PCs in AD

AD is a prevalent inflammatory skin disease, clinically manifesting as periodic episodes, intractable pruritus, and polymorphous eczematous skin lesions. Its etiology is multifactorial, mainly involving genetic susceptibility, immune dysregulation, skin barrier dysfunction and environmental triggers (Radhakrishnan et al., 2024; Patrizi et al., 2016). Although the precise pathogenesis remains incompletely understood, AD is widely considered as an immune-mediated disorder, featuring elevated serum IgE, Th1/Th2 cytokine imbalance (overactive Th2-dominant immune response), increased peripheral eosinophilia and extensive cutaneous ROS generation. Especially, the raised ROS in turn initiates OS that further destroys the barrier integrity and amplifies inflammatory responses in skin, consequently triggering AD occurrence (Dorjsembe et al., 2022; Park KH. et al., 2011). Meanwhile, Th2 cytokines can also act on sensory neurons via the JAK-STAT pathway to directly mediate chronic pruritus, forming an 'itch–scratch' cycle. The integrity of the skin barrier depends on the normal expression of stratum corneum structural proteins (such as filaggrin) and lipids. Patients with AD frequently carry filaggrin gene mutations or exhibit downregulated filaggrin expression, and Th2 inflammation (IL-4/IL-13) can further suppress filaggrin expression, establishing a vicious cycle (Howell et al., 2009).

In allergic inflammation models, oligomeric PCs have been shown to reduce serum IgE and Th2-type cytokine levels (e.g., IL-4) while alleviating eosinophil-associated inflammation, suggesting that they exert anti-inflammatory effects by suppressing the Th2 immune response (Zhou et al., 2011; Zhou et al., 2015). Given that AD is a predominantly Th2-driven immune-mediated inflammatory skin disease, these findings support the potential of PCs to attenuate the Th2-type inflammation characteristic of AD. In the *in vivo* experiments, we observed a phenomenon in an AD model induced by 20 weeks of mite antigen exposure, PCs-rich extract of Rosa multiflora THUNB. (RM) root significantly reduced antigen-driven eosinophilia and lesional IgE levels (Park et al., 2014). Similarly, other studies show PCs downregulate IgE receptor expression in ovalbumin-sensitized mice, thus lowering the body’s IgE (Takano et al., 2007). In addition, dietary grape, which is rich in PCs, conferred protection against AD-like skin lesions in NC/Nga mice (Singh et al., 2022). Furthermore, another animal mouse model of AD revealed that PCs also improved the skin barrier dysfunction caused by AD; they promote barrier repair by stimulating keratinocyte proliferation/differentiation and modulating lipid metabolism to increase cutaneous ceramide levels. Given that optimal ceramide concentrations are essential for moisture homeostasis, this dual mechanism effectively reinforces skin barrier function (Ikarashi et al., 2012; Ogawa and Yazaki, 2018). Since the onset of AD involves a complex network of Th2 inflammation, barrier damage, and OS, PCs, through multiple mechanisms such as anti-inflammatory, antioxidant, and immunomodulatory effects, are expected to have a synergistic intervention effect at different stages of the disease.

### 4.4 PCs in RA

RA is a chronic systemic inflammatory disease characterized by persistent synovitis and synovial hyperplasia, culminating in progressive joint destruction and disability. In pathogenesis, ROS drive disease progression by overwhelming the antioxidant defenses, activating inflammatory cascades, and oxidatively damaging cellular macromolecules to accelerate cartilage and bone destruction (Zhao et al., 2025; Jang et al., 2022). Central to RA pathophysiology are pro-inflammatory cytokines TNF-α and IL-1β. TNF-α regulates lymphocyte differentiation and promotes osteoclast-mediated bone resorption, while IL-1β encourages cartilage degradation. Both cytokines upregulate iNOS, ROS and COX-2, amplifying PGE2 and NO production to perpetuate inflammation and tissue injury (Sharma et al., 2014; Kiki et al., 2022). Besides, elevated toll-like receptor 4 (TLR4) expression in RA synovium correlates with persistent inflammation and joint destruction (Kim et al., 2018).

PCs attenuate articular inflammation through not only suppressing RA-related cytokines and inflammatory mediators, but also blocking synovial cell proliferation/migration and osteoclast differentiation. PCs-derived GSPE exhibits potent anti-inflammatory and cytoprotective effects. It has been verified in various animal models. Cho et al. first demonstrated GSPE’s therapeutic potential in collagen-induced arthritis (CIA) models, showing it ameliorates ROS and suppresses osteoclast differentiation *in vitro* (Cho et al., 2009). Subsequent studies confirmed that GSPE effectively prevents inflammation-mediated bone erosion through multi-targeted mechanisms: on the one hand, by inhibiting the NLRP3 inflammasome, a key molecular platform in the maturation and secretion of IL-1β, and blocking the NF-κB pathway, it can directly or indirectly reduce the gene transcription and protein expression of TNF-α and IL-1β, so as to effectively inhibit joint inflammation (Yin et al., 2022; Cai et al., 2016). Meanwhile, in the mouse model of synovitis used by Kim et al., mice treated with GSPE showed fewer TLR4-expressing cells in the synovium than untreated mice. This suggests that, *in vivo*, GSPE may exert its anti-arthritic effects by suppressing TLR4 expression in the synovium, thereby alleviating RA-associated inflammation and joint destruction (Kim et al., 2018; Qian et al., 2024). Similarly, Heo et al. found that GSPE induces apoptosis and cell autophagy in RA-fibroblast-like synoviocytes while inhibiting ROS generation, thereby controlling RA inflammation (H et al., 2022). IL-17, produced by Th17 cells, is crucial in autoimmune disease progression, specifically driving RA onset and advancement—evidenced by suppressed CIA pathology in IL-17-deficient mice (Kelepouri et al., 2018; Kugyelka et al., 2016). Nakamura et al. demonstrate that apple condensed tannins (ACT), rich in B1 and B2 type PCs, effectively suppress IL-17 expression to modulate RA inflammatory responses (Nakamura et al., 2015).

### 4.5 PCs in asthma

Bronchial asthma is a heterogeneous disease characterized by chronic airway inflammation, airway hyperresponsiveness (AHR), and reversible airflow limitation. Its immunopathology is centered around type 2 (Th2) inflammation. After exposure to allergens, antigen-presenting cells such as dendritic cells activate Th2 cells and type 2 innate lymphoid cells, which secrete cytokines such as IL-4, IL-5, and IL-13, driving a series of downstream events:IL-4 promotes B-cell isotype switching to IgE, IL-5 recruits and activates eosinophils, and IL-13 induces mucus hypersecretion, subepithelial fibrosis, and airway hyperresponsiveness (Hammad and Lambrecht, 2021). Recurrent chronic inflammation can lead to airway remodeling, manifested as airway smooth muscle hyperplasia, goblet cell metaplasia, and extracellular matrix deposition, ultimately resulting in irreversible airway dysfunction (Holgate, 2012; Hassoun et al., 2022). Furthermore, OS also plays an important role in the pathogenesis of asthma; during acute asthma exacerbations, the activity of SOD declines rapidly, suggesting that the weakening of pulmonary antioxidant defenses contributes to disease exacerbation (Comhair et al., 2000).

Multiple animal studies have shown that GSPE exert significant alleviating effects on allergic airway inflammation. In an ovalbumin-induced mouse model of asthma, GSPE attenuated the pathological changes and inflammatory injury of the lung parenchyma (Mahmoud, 2012). In another mouse model of chronic asthma, GSPE inhibited the infiltration of inflammatory cells (particularly eosinophils), reduced airway resistance, and suppressed the development of airway remodeling, indicating that PCs act on the key processes of inflammatory cell recruitment and structural remodeling in allergic airway inflammation (Zhou et al., 2015). GSPE has also been shown to attenuate airway inflammation and airway hyperresponsiveness by downregulating iNOS, suggesting that it suppresses the nitric oxide-related inflammatory amplification in asthmatic airways (Zhou et al., 2011). Moreover, given the role of OS in the onset and exacerbation of asthma, the potent free-radical-scavenging and antioxidant capacities of PCs are also expected to alleviate airway inflammation by enhancing pulmonary antioxidant defenses and reducing oxidative injury (Comhair et al., 2000).

### 4.6 Clinical evidence and translational challenges of PCs in IMIDs

Compared with the abundant preclinical evidence, human studies of PCs in IMIDs remain preliminary, although several noteworthy clinical explorations have emerged. In IBD, one study assessed the effect of dietary PCs intake on symptoms in UC patients in remission, suggesting an adjunctive value; a prospective pilot study at the University Hospital of Padua further evaluated an 8-week PCs treatment and found that it significantly improved the subjective quality of life of UC patients in clinical remission (Głąbska et al., 2020; Facchin et al., 2026). In osteoarthritis, a randomized, double-blind, placebo-controlled trial showed that pine bark extract (Pycnogenol), which is rich in oligomeric PCs, improved symptoms of mild-to-moderate knee osteoarthritis and reduced the use of nonsteroidal anti-inflammatory drugs (NSAIDs) (Cisár et al., 2008). Another study in osteoarthritis patients also observed that Pycnogenol reduced C-reactive protein (CRP), plasma free radicals, and fibrinogen levels, indicating that its clinical benefit was accompanied by improvements in systemic inflammatory markers (Belcaro et al., 2008). Regarding asthma, the latest research has confirmed that oligomeric proanthocyanidin can reduce the severity of asthma and inhibit airway remodeling. The mechanism involves regulating the interaction between epithelial and smooth muscle cells (Gu et al., 2025). In a 4-week open-label human trial, Sano reported that 29 healthy Japanese volunteers receiving daily oral doses of 1000, 1500, or 2500 mg of GSPE experienced no serious adverse events, providing direct evidence of the high-dose safety of GSPE in humans (Sano, 2017). Consistently, a systematic review of Pycnogenol, a French maritime pine bark extract rich in oligomeric PCs, concluded that it was essentially free of adverse effects and constitutes an effective and safe supplement (Weichmann and Rohdewald, 2024). Together, these findings further validate the favorable tolerability and safety of PCs and highlight their promising prospects in clinical application. It must be acknowledged, however, that despite abundant basic research, the clinical evidence for PCs in IMIDs remains very limited, reflecting a clear translational gap. The extremely low bioavailability of PCs constitutes the most fundamental obstacle to their progression from basic research to clinical application. As large polymeric molecules, PCs exhibit an absorption capacity closely linked to their DP, studies have unequivocally shown that only low-molecular-weight oligomers are absorbed intact in the gastrointestinal tract, while high-molecular-weight polymers dominate in foods (Rasmussen et al., 2005). Furthermore, their health effects rely heavily on gut microbiota metabolism, and given the marked inter-individual variation of the gut microbiota, different individuals display substantial differences in their responses to the same dietary component (Zeevi et al., 2015).

## 5 Conclusion

In summary, IMIDs represent a spectrum of chronic, disabling conditions characterized by systemic involvement and persistent inflammation driven by dysregulated immune activation. PCs, with their powerful anti-inflammatory and immunomodulatory properties, are emerging as promising natural therapeutic candidates that may offer multi-targeted efficacy with favorable safety profiles compared to conventional immunosuppressants for IMIDs. Therefore, through continued mechanistic elucidation and rigorous clinical validation, PCs hold promise as an innovative adjuvant strategy—potentially reducing reliance on synthetic immunomodulators while delivering substantial clinical benefits and quality-of-life improvements for IMIDs patients.

## References

[B1] Adelaja A. Hoffmann A. (2019). Signaling crosstalk mechanisms that May fine-tune pathogen-responsive NFκB. Front. Immunol. 10, 433. 10.3389/fimmu.2019.00433 31312197 PMC6614373

[B2] Ahmad S. F. Zoheir K. M. Abdel-Hamied H. E. Ashour A. E. Bakheet S. A. Attia S. M. (2013). Grape seed proanthocyanidin extract has potent anti-arthritic effects on collagen-induced arthritis by modifying the T cell balance. Int. Immunopharmacol. 17 (1), 79–87. 10.1016/j.intimp.2013.05.026 23759204

[B3] Alıravcı I. D. Mutlu P. Oymak S. Guney U. I. Keskin O. (2025). Risk of latent tuberculosis infection reactivation in patients treated with tumor necrosis factor antagonists: a five-year retrospective Study. Trop. Med. Infect. Dis. 10 (7), 190. 10.3390/tropicalmed10070190 PMC12300220 40711067

[B4] Appeldoorn M. M. Vincken J. P. Gruppen H. Hollman P. C. (2009). Procyanidin dimers A1, A2, and B2 are absorbed without conjugation or methylation from the small intestine of rats. J. Nutr. 139 (8), 1469–1473. 10.3945/jn.109.106765 19494022

[B5] Aron P. M. Kennedy J. A. (2008). Flavan-3-ols: nature, occurrence and biological activity. Mol. Nutr. Food Res. 52 (1), 79–104. 10.1002/mnfr.200700137 18081206

[B6] Bancil A. S. Sandall A. M. Rossi M. Chassaing B. Lindsay J. O. Whelan K. (2021). Food additive emulsifiers and their impact on Gut Microbiome, permeability, and inflammation: mechanistic insights in inflammatory bowel disease. J. Crohns Colitis 15 (6), 1068–1079. 10.1093/ecco-jcc/jjaa254 33336247

[B7] Belcaro G. Cesarone M. R. Errichi S. Zulli C. Errichi B. M. Vinciguerra G. (2008). Variations in C-reactive protein, plasma free radicals and fibrinogen values in patients with osteoarthritis treated with Pycnogenol. Redox Rep. 13 (6), 271–276. 10.1179/135100008X309019 19017467

[B8] Bellinger D. L. Lorton D. (2018). Sympathetic nerve hyperactivity in the spleen: causal for nonpathogenic-driven chronic immune-mediated inflammatory diseases (IMIDs)? Int. J. Mol. Sci. 19 (4), 1188. 10.3390/ijms19041188 29652832 PMC5979464

[B9] Bitzer Z. T. Glisan S. L. Dorenkott M. R. Goodrich K. M. Ye L. O'Keefe S. F. (2015). Cocoa procyanidins with different degrees of polymerization possess distinct activities in models of colonic inflammation. J. Nutr. Biochem. 26 (8), 827–831. 10.1016/j.jnutbio.2015.02.007 25869594 PMC4469546

[B10] Bladé C. Aragonès G. Arola-Arnal A. Muguerza B. Bravo F. I. Salvadó M. J. (2016). Proanthocyanidins in health and disease. Biofactors 42 (1), 5–12. 10.1002/biof.1249 26762288

[B11] Bohn T. (2014). Dietary factors affecting polyphenol bioavailability. Nutr. Rev. 72 (7), 429–452. 10.1111/nure.12114 24828476

[B12] Bovee T. F. Helsdingen R. J. Hoogenboom R. L. de Nijs M. W. Liu X. Vrieling K. (2015). Are effects of common ragwort in the Ames test caused by pyrrolizidine alkaloids? Mutat. Res. 778, 1–10. 10.1016/j.mrfmmm.2015.05.006 26021695

[B13] Brown M. A. Xu H. Li Z. (2020). Genetics and the axial spondyloarthritis spectrum. Rheumatol. Oxf. 59 (Suppl. 4), iv58–iv66. 10.1093/rheumatology/keaa464 33053195 PMC7566537

[B14] Bunte K. Beikler T. (2019). Th17 cells and the IL-23/IL-17 axis in the pathogenesis of Periodontitis and immune-mediated inflammatory diseases. Int. J. Mol. Sci. 20 (14), 3394. 10.3390/ijms20143394 31295952 PMC6679067

[B15] Burlec A. F. Hăncianu M. Ivănescu B. Macovei I. Corciovă A. (2024). Exploring the therapeutic potential of natural compounds in psoriasis and their inclusion in nanotechnological systems. Antioxidants (Basel) 13 (8), 912. 10.3390/antiox13080912 PMC11352172 39199158

[B16] Cai Y. Kong H. Pan Y. B. Jiang L. Pan X. X. Hu L. (2016). Procyanidins alleviates morphine tolerance by inhibiting activation of NLRP3 inflammasome in microglia. J. Neuroinflammation 13 (1), 53. 10.1186/s12974-016-0520-z 26931361 PMC4774188

[B17] Cai X. Han Y. Gu M. Song M. Wu X. Li Z. (2019). Dietary cranberry suppressed colonic inflammation and alleviated gut microbiota dysbiosis in dextran sodium sulfate-treated mice. Food Funct. 10 (10), 6331–6341. 10.1039/c9fo01537j 31524900 PMC6800821

[B18] Chan S. S. M. Chen Y. Casey K. Olen O. Ludvigsson J. F. Carbonnel F. (2022). Obesity is associated with increased risk of Crohn's disease, but not ulcerative colitis: a pooled analysis of five prospective cohort studies. Clin. Gastroenterol. Hepatol. 20 (5), 1048–1058. 10.1016/j.cgh.2021.06.049 34242756

[B19] Chen S. J. Lin G. J. Chen J. W. Wang K. C. Tien C. H. Hu C. F. (2019). Immunopathogenic mechanisms and novel immune-modulated therapies in rheumatoid arthritis. Int. J. Mol. Sci. 20 (6), 1332. 10.3390/ijms20061332 30884802 PMC6470801

[B20] Chen B. Sun L. Zeng G. Shen Z. Wang K. Yin L. (2022). Gut bacteria alleviate smoking-related NASH by degrading gut nicotine. Nature 610 (7932), 562–568. 10.1038/s41586-022-05299-4 PMC9589931 36261549

[B21] Cho M. L. Heo Y. J. Park M. K. Oh H. J. Park J. S. Woo Y. J. (2009). Grape seed proanthocyanidin extract (GSPE) attenuates collagen-induced arthritis. Immunol. Lett. 124 (2), 102–110. 10.1016/j.imlet.2009.05.001 19446580

[B22] Choy Y. Y. Jaggers G. K. Oteiza P. I. Waterhouse A. L. (2013). Bioavailability of intact proanthocyanidins in the rat colon after ingestion of grape seed extract. J. Agric. Food Chem. 61 (1), 121–127. 10.1021/jf301939e 23244439

[B23] Chu H. Tang Q. Huang H. Hao W. Wei X. (2016). Grape-seed proanthocyanidins inhibit the lipopolysaccharide-induced inflammatory mediator expression in RAW264.7 macrophages by suppressing MAPK and NF-κb signal pathways. Environ. Toxicol. Pharmacol. 41, 159–166. 10.1016/j.etap.2015.11.018 26708200

[B24] Cisár P. Jány R. Waczulíková I. Sumegová K. Muchová J. Vojtassák J. (2008). Effect of pine bark extract (Pycnogenol) on symptoms of knee osteoarthritis. Phytother. Res. 22 (8), 1087–1092. 10.1002/ptr.2461 18570266

[B25] Comhair S. A. Bhathena P. R. Dweik R. A. Kavuru M. Erzurum S. C. (2000). Rapid loss of superoxide dismutase activity during antigen-induced asthmatic response. Lancet 355, 624. 10.1016/S0140-6736(99)04736-4 10696986

[B26] Cush J. J. (2022). Rheumatoid arthritis: early diagnosis and treatment. Rheum. Dis. Clin. North Am. 48 (2), 537–547. 10.1016/j.rdc.2022.02.010 35400377

[B27] Cutolo M. Sulli A. Paolino S. Pizzorni C. (2016). CTLA-4 blockade in the treatment of rheumatoid arthritis: an update. Expert Rev. Clin. Immunol. 12 (4), 417–425. 10.1586/1744666X.2016.1133295 26679631

[B28] Das T. Chatterjee N. Capanoglu E. Lorenzo J. M. Das A. K. Dhar P. (2023). The synergistic ramification of insoluble dietary fiber and associated non-extractable polyphenols on gut microbial population escorting alleviation of lifestyle diseases. Food Chem. X 18, 100697. 10.1016/j.fochx.2023.100697 37206320 PMC10189415

[B29] David T. Ling S. F. Barton A. (2018). Genetics of immune-mediated inflammatory diseases. Clin. Exp. Immunol. 193 (1), 3–12. 10.1111/cei.13101 29328507 PMC6037997

[B30] De Santis S. Kunde D. Galleggiante V. Liso M. Scandiffio L. Serino G. (2017). TNFα deficiency results in increased IL-1β in an early onset of spontaneous murine colitis. Cell Death Dis. 8 (8), e2993. 10.1038/cddis.2017.397 28796256 PMC5596580

[B31] Dendrou C. A. Petersen J. Rossjohn J. Fugger L. (2018). HLA variation and disease. Nat. Rev. Immunol. 18 (5), 325–339. 10.1038/nri.2017.143 29292391

[B32] Diaz M. S. Mertens-Talcott S. U. Talcott S. T. (2024). Intestinal microbiome metabolism of cranberry (Vaccinium macrocarpon) proanthocyanidin dimers, but not trimers, is altered by dysbiosis in ulcerative colitis *Ex Vivo* . J. Agric. Food Chem. 72 (8), 4184–4194. 10.1021/acs.jafc.4c00042 38350030 PMC10905997

[B33] Dong C. (2008). TH17 cells in development: an updated view of their molecular identity and genetic programming. Nat. Rev. Immunol. 8 (5), 337–348. 10.1038/nri2295 18408735

[B34] Dorjsembe B. Nho C. W. Choi Y. Kim J. C. (2022). Extract from Black soybean cultivar A63 extract ameliorates atopic dermatitis-like skin inflammation in an oxazolone-induced murine model. Molecules 27 (9), 2751. 10.3390/molecules27092751 35566102 PMC9104407

[B35] Facchin S. Agostini E. Laparra-Ruiz E. Benvenuto G. Valle G. Bertin L. (2026). Protective effects of grapeseed proanthocyanidins in ulcerative colitis: a pilot Study evaluating a potential therapeutic strategy. J. Clin. Med. 15 (2), 888. 10.3390/jcm15020888 41598825 PMC12841892

[B36] García-Domínguez M. (2025). The role of IL-23 in the development of inflammatory diseases. Biol. (Basel) 14 (4), 347. 10.3390/biology14040347 PMC12025033 40282212

[B37] García-Pérez M. E. Royer M. Duque-Fernandez A. Diouf P. N. Stevanovic T. Pouliot R. (2010). Antioxidant, toxicological and antiproliferative properties of Canadian polyphenolic extracts on normal and psoriatic keratinocytes. J. Ethnopharmacol. 132 (1), 251–258. 10.1016/j.jep.2010.08.030 20727399

[B38] Głąbska D. Guzek D. Gałązka K. Lech G. (2020). Therapeutic potential of proanthocyanidins in ulcerative colitis in remission. J. Clin. Med. 9 (3), 771. 10.3390/jcm9030771 32178368 PMC7141196

[B39] Global, regional (2023). Global, regional, and national incidence of six major immune-mediated inflammatory diseases: findings from the global burden of disease study 2019. EClinicalMedicine. 64, 102193. 10.1016/j.eclinm.2023.102193 37731935 PMC10507198

[B40] Gómez-Bañuelos E. Mukherjee A. Darrah E. Andrade F. (2019). Rheumatoid arthritis-associated mechanisms of Porphyromonas gingivalis and Aggregatibacter actinomycetemcomitans. J. Clin. Med. 8 (9), 1309. 10.3390/jcm8091309 31454946 PMC6780899

[B41] González-Quilen C. Rodríguez-Gallego E. Beltrán-Debón R. Pinent M. Ardévol A. Blay M. T. (2020). Health-Promoting properties of proanthocyanidins for intestinal dysfunction. Nutrients 12 (1), 130. 10.3390/nu12010130 31906505 PMC7019584

[B42] Gregersen P. K. Silver J. Winchester R. J. (1987). The shared epitope hypothesis. An approach to understanding the molecular genetics of susceptibility to rheumatoid arthritis. Arthritis Rheum. 30 (11), 1205–1213. 10.1002/art.1780301102 2446635

[B43] Griffiths C. E. M. Armstrong A. W. Gudjonsson J. E. Barker J. (2021). Psoriasis. Lancet 397 (10281), 1301–1315. 10.1016/s0140-6736(20)32549-6 33812489

[B44] Gu S. Gu Y. Gu H. Zhong X. Liang W. Liu Y. (2025). Oligomeric proanthocyanidin attenuates asthma severity and airway remodeling by modulating epithelial-smooth muscle cell interactions. Phytomedicine 142, 156798. 10.1016/j.phymed.2025.156798 40347887

[B45] Gudjonsson J. E. Thorarinsson A. M. Sigurgeirsson B. Kristinsson K. G. Valdimarsson H. (2003). Streptococcal throat infections and exacerbation of chronic plaque psoriasis: a prospective study. Br. J. Dermatol 149 (3), 530–534. 10.1046/j.1365-2133.2003.05552.x 14510985

[B46] Guo J. Zhang H. Lin W. Lu L. Su J. Chen X. (2023). Signaling pathways and targeted therapies for psoriasis. Signal Transduct. Target Ther. 8 (1), 437. 10.1038/s41392-023-01655-6 38008779 PMC10679229

[B47] Heo Y. R. Son C. N. Baek W. K. Kim S. H. (2022). Grape seed proanthocyanidin extract induces apoptotic and autophagic cell death in rheumatoid arthritis fibroblast-like synoviocytes. Arch. Rheumatol. 37 (3), 393–403. 10.46497/archrheumatol.2022.9034 36589610 PMC9791554

[B48] Hah Y. S. Kim J. G. Cho H. Y. Park J. S. Heo E. P. Yoon T. J. (2017). Procyanidins from Vitis vinifera seeds induce apoptotic and autophagic cell death *via* generation of reactive oxygen species in squamous cell carcinoma cells. Oncol. Lett. 14 (2), 1925–1932. 10.3892/ol.2017.6422 28781636 PMC5530194

[B49] Hammad H. Lambrecht B. N. (2021). The basic immunology of asthma. Cell 184 (6), 1469–1485. 10.1016/j.cell.2021.02.016 33711259

[B50] Han S. Gao H. Chen S. Wang Q. Li X. Du L. J. (2019). Procyanidin A1 Alleviates Inflammatory Response induced by LPS through NF-κB, MAPK, and Nrf2/HO-1 Pathways in RAW264.7 cells. Sci. Rep. 9 (1), 15087. 10.1038/s41598-019-51614-x 31636354 PMC6803657

[B51] Hartwig T. Zwicky P. Schreiner B. Yawalkar N. Cheng P. Navarini A. (2018). Regulatory T cells restrain pathogenic T helper cells during skin inflammation. Cell Rep. 25 (13), 3564. 10.1016/j.celrep.2018.12.012 30590032

[B52] Hassoun D. Rose L. Blanc F. X. Magnan A. Loirand G. Sauzeau V. (2022). Bronchial smooth muscle cell in asthma: where does it fit? BMJ Open Respir. Res. 9 (1), e001351. 10.1136/bmjresp-2022-001351 PMC9478857 36109087

[B53] He J. Guo H. Zou M. (2025). Research advances in nanomaterials with aromatic functional groups for the treatment of inflammatory bowel disease. Front. Bioeng. Biotechnol. 13, 1614939. 10.3389/fbioe.2025.1614939 40630942 PMC12234557

[B54] Helbig K. L. Nothnagel M. Hampe J. Balschun T. Nikolaus S. Schreiber S. (2012). A case-only study of gene-environment interaction between genetic susceptibility variants in NOD2 and cigarette smoking in Crohn's disease aetiology. BMC Med. Genet. 13, 14. 10.1186/1471-2350-13-14 22416979 PMC3314543

[B55] Higgins E. (2000). Alcohol, smoking and psoriasis. Clin. Exp. Dermatol 25 (2), 107–110. 10.1046/j.1365-2230.2000.00588.x 10733631

[B56] Holgate S. T. (2012). Innate and adaptive immune responses in asthma. Nat. Med. 18 (5), 673–683. 10.1038/nm.2731 22561831

[B57] Howell A. B. Reed J. D. Krueger C. G. Winterbottom R. Cunningham D. G. Leahy M. (2005). A-type cranberry proanthocyanidins and uropathogenic bacterial anti-adhesion activity. Phytochemistry 66 (18), 2281–2291. 10.1016/j.phytochem.2005.05.022 16055161

[B58] Howell M. D. Kim B. E. Gao P. Grant A. V. Boguniewicz M. DeBenedetto A. (2009). Cytokine modulation of atopic dermatitis filaggrin skin expression. J. Allergy Clin. Immunol. 124 (3 Suppl. 2), R7–r12. 10.1016/j.jaci.2009.07.012 19720210 PMC13237620

[B59] Hu Y. Yang Y. Li Y. Zhang Q. Zhang W. Jia J. (2025). Th17/Treg imbalance in inflammatory bowel disease: immunological mechanisms and microbiota-driven regulation. Front. Immunol. 16, 1651063. 10.3389/fimmu.2025.1651063 41132656 PMC12540096

[B60] Hugot J. P. Chamaillard M. Zouali H. Lesage S. Cézard J. P. Belaiche J. (2001). Association of NOD2 leucine-rich repeat variants with susceptibility to Crohn's disease. Nature 411 (6837), 599–603. 10.1038/35079107 11385576

[B61] Ikarashi N. Sato W. Toda T. Ishii M. Ochiai W. Sugiyama K. (2012). Inhibitory effect of polyphenol-rich fraction from the bark of Acacia mearnsii on itching associated with allergic dermatitis. Evid. Based Complement. Altern. Med. 2012, 120389. 10.1155/2012/120389 22315629 PMC3270726

[B62] Ishigaki K. Lagattuta K. A. Luo Y. James E. A. Buckner J. H. Raychaudhuri S. (2022). HLA autoimmune risk alleles restrict the hypervariable region of T cell receptors. Nat. Genet. 54 (4), 393–402. 10.1038/s41588-022-01032-z 35332318 PMC9010379

[B63] Jang S. Kwon E. J. Lee J. J. (2022). Rheumatoid arthritis: pathogenic roles of diverse immune cells. Int. J. Mol. Sci. 23 (2), 905. 10.3390/ijms23020905 PMC8780115 35055087

[B64] Jonker A. Yu P. (2017). The occurrence, biosynthesis, and molecular structure of proanthocyanidins and their effects on Legume forage protein precipitation, digestion and absorption in the ruminant digestive tract. Int. J. Mol. Sci. 18 (5), 1105. 10.3390/ijms18051105 28531145 PMC5455013

[B65] Kelepouri D. Mavropoulos A. Bogdanos D. P. Sakkas L. I. (2018). The role of flavonoids in inhibiting Th17 responses in inflammatory arthritis. J. Immunol. Res. 2018, 9324357. 10.1155/2018/9324357 29693024 PMC5859886

[B66] Kerdidani D. Papaioannou N. E. Nakou E. Alissafi T. (2022). Rebooting regulatory T cell and dendritic cell function in immune-mediated inflammatory diseases: biomarker and therapy discovery under a multi-omics lens. Biomedicines 10 (9), 2140. 10.3390/biomedicines10092140 36140240 PMC9495698

[B67] Kiki G. Pop R. M. Sabin O. Bocsan I. C. Chedea V. S. Socaci S. A. (2022). Polyphenols from Dichrostachys cinerea fruits Anti-Inflammatory, analgesic, and antioxidant capacity in freund's adjuvant-induced arthritic rat model. Molecules 27 (17), 5445. 10.3390/molecules27175445 36080212 PMC9457916

[B68] Kim S. H. Bang J. Son C. N. Baek W. K. Kim J. M. (2018). Grape seed proanthocyanidin extract ameliorates murine autoimmune arthritis through regulation of TLR4/MyD88/NF-κB signaling pathway. Korean J. Intern Med. 33 (3), 612–621. 10.3904/kjim.2016.053 27271273 PMC5943648

[B69] Kuek A. Hazleman B. L. Ostör A. J. (2007). Immune-mediated inflammatory diseases (IMIDs) and biologic therapy: a medical revolution. Postgrad. Med. J. 83 (978), 251–260. 10.1136/pgmj.2006.052688 17403952 PMC2600039

[B70] Kugyelka R. Kohl Z. Olasz K. Mikecz K. Rauch T. A. Glant T. T. (2016). Enigma of IL-17 and Th17 cells in rheumatoid arthritis and in autoimmune animal models of arthritis. Mediat. Inflamm. 2016, 6145810. 10.1155/2016/6145810 26903711 PMC4745575

[B71] Lai R. Xian D. Xiong X. Yang L. Song J. Zhong J. (2018). Proanthocyanidins: novel treatment for psoriasis that reduces oxidative stress and modulates Th17 and Treg cells. Redox Rep. 23 (1), 130–135. 10.1080/13510002.2018.1462027 29630472 PMC6748681

[B72] Laudisi F. Di Fusco D. Dinallo V. Stolfi C. Di Grazia A. Marafini I. (2019). The food Additive Maltodextrin promotes endoplasmic reticulum stress-driven mucus depletion and exacerbates intestinal inflammation. Cell Mol. Gastroenterol. Hepatol. 7 (2), 457–473. 10.1016/j.jcmgh.2018.09.002 30765332 PMC6369223

[B73] Lee S. Y. Yoon B. Y. Kim J. I. Heo Y. M. Woo Y. J. Park S. H. (2014). Interleukin-17 increases the expression of toll-like receptor 3 *via* the STAT3 pathway in rheumatoid arthritis fibroblast-like synoviocytes. Immunology 141 (3), 353–361. 10.1111/imm.12196 24708416 PMC3930374

[B74] Li Y. Yu Q. Zhao W. Zhang J. Liu W. Huang M. (2017). Oligomeric proanthocyanidins attenuate airway inflammation in asthma by inhibiting dendritic cells maturation. Mol. Immunol. 91, 209–217. 10.1016/j.molimm.2017.09.012 28963930

[B75] Li L. Huang X. Cheng M. He C. Dai C. Ding F. (2025). Pulmonary hypertension: etiology and anti-inflammatory treatment pathways of natural products. Front. Pharmacol. 16, 1743782. 10.3389/fphar.2025.1743782 41668810 PMC12883753

[B76] Lim I. Ha J. (2021). Biosynthetic pathway of proanthocyanidins in major cash crops. Plants (Basel) 10 (9), 1792. 10.3390/plants10091792 PMC8472070 34579325

[B77] Liu H. Y. Yuan P. Li S. Ogamune K. J. Shi X. Zhu C. (2025). Lactobacillus johnsonii alleviates experimental colitis by restoring intestinal barrier function and reducing NET-mediated gut-liver inflammation. Commun. Biol. 8 (1), 1222. 10.1038/s42003-025-08679-4 40813467 PMC12354853

[B78] Long D. Mao C. Huang Y. Xu Y. Zhu Y. (2024). Ferroptosis in ulcerative colitis: potential mechanisms and promising therapeutic targets. Biomed. Pharmacother. 175, 116722. 10.1016/j.biopha.2024.116722 38729051

[B79] Lopetuso L. R. Murgiano M. Mantuano E. Schiavone V. Costa A. Mascianà G. (2025). The molecular landscape of inflammation in Inflammatory Bowel Disease (IBD): targets for precision medicine. Biomedicines 13 (11), 2738. 10.3390/biomedicines13112738 41301831 PMC12650290

[B80] López-Ferrer A. Laiz A. Puig L. (2022). Psoriatic arthritis. Med. Clin. Barc. 159 (1), 40–46. 10.1016/j.medcle.2022.01.013 35525675

[B81] Lu Q. Ye Z. Yang C. (2025). Optimization of ultrasonic-enzyme synergistic extraction of proanthocyanidins from jujube: purification, characterization, and bioactivity Study. Molecules 30 (3), 619. 10.3390/molecules30030619 39942723 PMC11820555

[B82] Luo P. Wang P. Xu J. Hou W. Xu P. Xu K. (2022). Immunomodulatory role of T helper cells in rheumatoid arthritis: a comprehensive research review. Bone Jt. Res. 11 (7), 426–438. 10.1302/2046-3758.117.bjr-2021-0594.r1 PMC9350707 35775145

[B83] Ma Z. F. Zhang H. (2017). Phytochemical constituents, health benefits, and industrial applications of grape seeds: a mini-review. Antioxidants (Basel) 6 (3), 71. 10.3390/antiox6030071 28914789 PMC5618099

[B84] Mahmoud Y. I. (2012). Grape seed extract attenuates lung parenchyma pathology in ovalbumin-induced mouse asthma model: an ultrastructural study. Micron 43 (10), 1050–1059. 10.1016/j.micron.2012.04.014 22609098

[B85] Mannino G. Chinigò G. Serio G. Genova T. Gentile C. Munaron L. (2021). Proanthocyanidins and where to find them: a meta-analytic approach to investigate their chemistry, biosynthesis, distribution, and effect on human health. Antioxidants (Basel) 10 (8), 1229. 10.3390/antiox10081229 34439477 PMC8389005

[B86] Mateos-Martín M. L. Fuguet E. Quero C. Pérez-Jiménez J. Torres J. L. (2012). New identification of proanthocyanidins in cinnamon (Cinnamomum zeylanicum L.) using MALDI-TOF/TOF mass spectrometry. Anal. Bioanal. Chem. 402 (3), 1327–1336. 10.1007/s00216-011-5557-3 22101466

[B87] McAleer J. P. Kolls J. K. (2011). Mechanisms controlling Th17 cytokine expression and host defense. J. Leukoc. Biol. 90 (2), 263–270. 10.1189/jlb.0211099 21486905 PMC3133441

[B88] McGeachy M. J. Chen Y. Tato C. M. Laurence A. Joyce-Shaikh B. Blumenschein W. M. (2009). The interleukin 23 receptor is essential for the terminal differentiation of interleukin 17-producing effector T helper cells *in vivo* . Nat. Immunol. 10 (3), 314–324. 10.1038/ni.1698 19182808 PMC2945605

[B89] McInnes I. B. Gravallese E. M. (2021). Immune-mediated inflammatory disease therapeutics: past, present and future. Nat. Rev. Immunol. 21 (10), 680–686. 10.1038/s41577-021-00603-1 34518662 PMC8436867

[B90] Mena P. Bresciani L. Brindani N. Ludwig I. A. Pereira-Caro G. Angelino D. (2019). Phenyl-γ-valerolactones and phenylvaleric acids, the main colonic metabolites of flavan-3-ols: synthesis, analysis, bioavailability, and bioactivity. Nat. Prod. Rep. 36 (5), 714–752. 10.1039/c8np00062j 30468210

[B91] Menter A. Krueger G. G. Paek S. Y. Kivelevitch D. Adamopoulos I. E. Langley R. G. (2021). Interleukin-17 and Interleukin-23: a narrative review of mechanisms of action in psoriasis and associated comorbidities. Dermatol Ther. (Heidelb) 11 (2), 385–400. 10.1007/s13555-021-00483-2 33512665 PMC8019008

[B92] Miller R. J. Jackson K. G. Dadd T. Nicol B. Dick J. L. Mayes A. E. (2012). A preliminary investigation of the impact of catechol-O-methyltransferase genotype on the absorption and metabolism of green tea catechins. Eur. J. Nutr. 51 (1), 47–55. 10.1007/s00394-011-0189-0 21445620

[B93] Miranda-Hernández A. M. Muñiz-Márquez D. B. Wong-Paz J. E. Aguilar-Zárate P. de la Rosa-Hernández M. Larios-Cruz R. (2019). Characterization by HPLC-ESI-MS(2) of native and oxidized procyanidins from litchi (Litchi chinensis) pericarp. Food Chem. 291, 126–131. 10.1016/j.foodchem.2019.04.020 31006450

[B94] Molinar-Toribio E. Ramos-Romero S. Fuguet E. Taltavull N. Méndez L. Romeu M. (2017). Influence of omega-3 PUFAs on the metabolism of proanthocyanidins in rats. Food Res. Int. 97, 133–140. 10.1016/j.foodres.2017.03.046 28578033

[B95] Monagas M. Urpi-Sarda M. Sánchez-Patán F. Llorach R. Garrido I. Gómez-Cordovés C. (2010). Insights into the metabolism and microbial biotransformation of dietary flavan-3-ols and the bioactivity of their metabolites. Food Funct. 1 (3), 233–253. 10.1039/c0fo00132e 21776473

[B96] Monteleone G. Moscardelli A. Colella A. Marafini I. Salvatori S. (2023). Immune-mediated inflammatory diseases: common and different pathogenic and clinical features. Autoimmun. Rev. 22 (10), 103410. 10.1016/j.autrev.2023.103410 37597601

[B97] Muñoz-Martínez C. Segura-Puertas M. Gómez-Moreno G. (2021). Disease-Modifying Antirheumatic Drugs (DMARDs) and drug interactions in dentistry. Eur. Rev. Med. Pharmacol. Sci. 25 (7), 2834–2842. 10.26355/eurrev_202104_25536 33877648

[B98] Nakamura K. Matsuoka H. Nakashima S. Kanda T. Nishimaki-Mogami T. Akiyama H. (2015). Oral administration of apple condensed tannins delays rheumatoid arthritis development in mice *via* downregulation of T helper 17 (Th17) cell responses. Mol. Nutr. Food Res. 59 (7), 1406–1410. 10.1002/mnfr.201400909 25917233

[B99] Nakano N. Nishiyama C. Tokura T. Nagasako-Akazome Y. Ohtake Y. Okumura K. (2008). Procyanidin C1 from apple extracts inhibits Fc epsilon RI-mediated mast cell activation. Int. Arch. Allergy Immunol. 147 (3), 213–221. 10.1159/000142044 18594151

[B100] Nandakumar V. Singh T. Katiyar S. K. (2008). Multi-targeted prevention and therapy of cancer by proanthocyanidins. Cancer Lett. 269 (2), 378–387. 10.1016/j.canlet.2008.03.049 18457915 PMC2562893

[B101] Navarro-Compán V. Puig L. Vidal S. Ramírez J. Llamas-Velasco M. Fernández-Carballido C. (2023). The paradigm of IL-23-independent production of IL-17F and IL-17A and their role in chronic inflammatory diseases. Front. Immunol. 14, 1191782. 10.3389/fimmu.2023.1191782 37600764 PMC10437113

[B102] Nicolaides S. Vasudevan A. Long T. van Langenberg D. (2021). The impact of tobacco smoking on treatment choice and efficacy in inflammatory bowel disease. Intest. Res. 19 (2), 158–170. 10.5217/ir.2020.00008 33040518 PMC8100381

[B103] Nie H. Zheng Y. Li R. Guo T. B. He D. Fang L. (2013). Phosphorylation of FOXP3 controls regulatory T cell function and is inhibited by TNF-α in rheumatoid arthritis. Nat. Med. 19 (3), 322–328. 10.1038/nm.3085 23396208

[B104] Nie F. Liu L. Cui J. Zhao Y. Zhang D. Zhou D. (2023). Oligomeric proanthocyanidins: an updated review of their natural sources, synthesis, and potentials. Antioxidants (Basel) 12 (5), 1004. 10.3390/antiox12051004 37237870 PMC10215713

[B105] Ogawa S. Yazaki Y. (2018). Tannins from Acacia mearnsii De Wild. Bark: tannin Determination and Biological Activities. Molecules 23 (4), 837. 10.3390/molecules23040837 29621196 PMC6017853

[B106] Ogulur I. Mitamura Y. Yazici D. Pat Y. Ardicli S. Li M. (2025). Type 2 immunity in allergic diseases. Cell Mol. Immunol. 22 (3), 211–242. 10.1038/s41423-025-01261-2 PMC11868591 39962262

[B107] Park M. K. Park J. S. Cho M. L. Oh H. J. Heo Y. J. Woo Y. J. (2011a). Grape seed proanthocyanidin extract (GSPE) differentially regulates Foxp3(+) regulatory and IL-17(+) pathogenic T cell in autoimmune arthritis. Immunol. Lett. 135 (1-2), 50–58. 10.1016/j.imlet.2010.09.011 20933009

[B108] Park K. H. Choi S. E. Choi Y. W. Lee D. I. Joo S. S. Jeong M. S. (2011b). Topical application of two condensed tannins from the root of Rosa multiflora Thunberg for the treatment of atopic dermatitis (AD) in NC/Nga mice. Phytother. Res. 25 (10), 1564–1569. 10.1002/ptr.3578 21717518

[B109] Park K. H. Jeong M. S. Park K. J. Choi Y. W. Seo S. J. Lee M. W. (2014). Topical application of Rosa multiflora root extract improves atopic dermatitis-like skin lesions induced by mite antigen in NC/Nga mice. Biol. Pharm. Bull. 37 (1), 178–183. 10.1248/bpb.b13-00619 24389494

[B110] Park J. Park H. Lee Y. L. Weon S. Kim Y. G. Yang J. H. (2021). Blocking TNFα attenuates progressive cartilage matrix degradation in inflammatory arthritis. Exp. Ther. Med. 22 (2), 808. 10.3892/etm.2021.10240 PMC8170641 34093764

[B111] Patrizi A. Raone B. Neri I. Gurioli C. Carbonara M. Cassano N. (2016). Randomized, controlled, double-blind clinical study evaluating the safety and efficacy of MD2011001 cream in mild-to-moderate atopic dermatitis of the face and neck in children, adolescents and adults. J. Dermatol. Treat. 27 (4), 346–350. 10.3109/09546634.2015.1115814 26652026

[B112] Picchianti-Diamanti A. Spinelli F. R. Rosado M. M. Conti F. Laganà B. (2021). Inhibition of Phosphodiesterase-4 in psoriatic arthritis and inflammatory bowel diseases. Int. J. Mol. Sci. 22 (5), 2638. 10.3390/ijms22052638 33807944 PMC7961737

[B113] Qian H. Su L. Yang Y. Tian X. Dai Q. Meng F. (2024). Selection and mechanism Study of Q-Markers for Xanthocerais lignum anti-rheumatoid arthritis based on serum spectrum-effect correlation analysis. Molecules 29 (13), 3191. 10.3390/molecules29133191 38999143 PMC11242948

[B114] Radhakrishnan J. Kennedy B. E. Noftall E. B. Giacomantonio C. A. Rupasinghe H. P. V. (2024). Recent advances in phytochemical-based topical applications for the management of eczema: a review. Int. J. Mol. Sci. 25 (10), 5375. 10.3390/ijms25105375 38791412 PMC11120771

[B115] Rasmussen S. E. Frederiksen H. Struntze Krogholm K. Poulsen L. (2005). Dietary proanthocyanidins: occurrence, dietary intake, bioavailability, and protection against cardiovascular disease. Mol. Nutr. Food Res. 49 (2), 159–174. 10.1002/mnfr.200400082 15635686

[B116] Rauf A. Imran M. Abu-Izneid T. Iahtisham Ul H. Patel S. Pan X. (2019). Proanthocyanidins: a comprehensive review. Biomed. Pharmacother. 116, 108999. 10.1016/j.biopha.2019.108999 31146109

[B117] Raza A. Yousaf W. Giannella R. Shata M. T. (2012). Th17 cells: interactions with predisposing factors in the immunopathogenesis of inflammatory bowel disease. Expert Rev. Clin. Immunol. 8 (2), 161–168. 10.1586/eci.11.96 22288454 PMC3937806

[B118] Rios L. Y. Bennett R. N. Lazarus S. A. Rémésy C. Scalbert A. Williamson G. (2002). Cocoa procyanidins are stable during gastric transit in humans. Am. J. Clin. Nutr. 76 (5), 1106–1110. 10.1093/ajcn/76.5.1106 12399286

[B119] Rodríguez-Pérez C. García-Villanova B. Guerra-Hernández E. Verardo V. (2019). Grape seeds proanthocyanidins: an overview of *in vivo* bioactivity in animal models. Nutrients 11 (10), 2435. 10.3390/nu11102435 31614852 PMC6835351

[B120] Round J. L. Mazmanian S. K. (2009). The gut microbiota shapes intestinal immune responses during health and disease. Nat. Rev. Immunol. 9 (5), 313–323. 10.1038/nri2515 19343057 PMC4095778

[B121] Sachen K. L. Hammaker D. Sarabia I. Stoveken B. Hartman J. Leppard K. L. (2025). Guselkumab binding to CD64(+) IL-23-producing myeloid cells enhances potency for neutralizing IL-23 signaling. Front. Immunol. 16, 1532852. 10.3389/fimmu.2025.1532852 40145093 PMC11937023

[B122] Sahoo D. K. Heilmann R. M. Paital B. Patel A. Yadav V. K. Wong D. (2023). Oxidative stress, hormones, and effects of natural antioxidants on intestinal inflammation in inflammatory bowel disease. Front. Endocrinol. (Lausanne) 14, 1217165. 10.3389/fendo.2023.1217165 37701897 PMC10493311

[B123] Sano A. (2017). Safety assessment of 4-week oral intake of proanthocyanidin-rich grape seed extract in healthy subjects. Food Chem. Toxicol. 108 (Pt B), 519–523. 10.1016/j.fct.2016.11.021 27889390

[B124] Scher J. U. Sczesnak A. Longman R. S. Segata N. Ubeda C. Bielski C. (2013). Expansion of intestinal Prevotella copri correlates with enhanced susceptibility to arthritis. Elife 2, e01202. 10.7554/eLife.01202 24192039 PMC3816614

[B125] Scherlinger M. Richez C. Tsokos G. C. Boilard E. Blanco P. (2023). The role of platelets in immune-mediated inflammatory diseases. Nat. Rev. Immunol. 23 (8), 495–510. 10.1038/s41577-023-00834-4 36707719 PMC9882748

[B126] Schinocca C. Rizzo C. Fasano S. Grasso G. La Barbera L. Ciccia F. (2021). Role of the IL-23/IL-17 pathway in rheumatic diseases: an overview. Front. Immunol. 12, 637829. 10.3389/fimmu.2021.637829 33692806 PMC7937623

[B127] Sharma S. Gupta R. Thakur S. C. (2014). Attenuation of collagen induced arthritis by Centella asiatica methanol fraction *via* modulation of cytokines and oxidative stress. Biomed. Environ. Sci. 27 (12), 926–938. 10.3967/bes2014.133 25484009

[B128] Shi Y. Zhang H. Li S. Xin D. Li S. Yan B. (2023). Procyanidin improves experimental colitis by regulating macrophage polarization. Biomed. Pharmacother. 165, 115076. 10.1016/j.biopha.2023.115076 37478578

[B129] Shin S. K. Cho J. H. Kim E. J. Kim E. K. Park D. K. Kwon K. A. (2017). Anti-inflammatory and anti-apoptotic effects of rosuvastatin by regulation of oxidative stress in a dextran sulfate sodium-induced colitis model. World J. Gastroenterol. 23 (25), 4559–4568. 10.3748/wjg.v23.i25.4559 28740344 PMC5504371

[B130] Shuhua Y. Lingqi M. Yunlong D. He T. Yao S. Peng L. (2022). Proanthocyanidins activate Nrf2/ARE signaling pathway in intestinal epithelial cells by inhibiting the ubiquitinated degradation of Nrf2. Biomed. Res. Int. 2022, 8562795. 10.1155/2022/8562795 PMC9410805 36033575

[B131] Singh C. K. Mintie C. A. Ndiaye M. A. Chhabra G. Roy S. Sullivan R. (2022). Protective effects of dietary grape against atopic dermatitis-like skin lesions in NC/NgaTndCrlj mice. Front. Immunol. 13, 1051472. 10.3389/fimmu.2022.1051472 36741360 PMC9893861

[B132] Smookler D. S. Mohammed F. F. Kassiri Z. Duncan G. S. Mak T. W. Khokha R. (2006). Tissue inhibitor of metalloproteinase 3 regulates TNF-dependent systemic inflammation. J. Immunol. 176 (2), 721–725. 10.4049/jimmunol.176.2.721 16393953

[B133] Sokol H. Pigneur B. Watterlot L. Lakhdari O. Bermúdez-Humarán L. G. Gratadoux J. J. (2008). Faecalibacterium prausnitzii is an anti-inflammatory commensal bacterium identified by gut microbiota analysis of Crohn disease patients. Proc. Natl. Acad. Sci. U. S. A. 105 (43), 16731–16736. 10.1073/pnas.0804812105 18936492 PMC2575488

[B134] Stark J. M. Tibbitt C. A. Coquet J. M. (2019). The metabolic requirements of Th2 Cell Differentiation. Front. Immunol. 10, 2318. 10.3389/fimmu.2019.02318 31611881 PMC6776632

[B135] Sunzini F. McInnes I. Siebert S. (2020). JAK inhibitors and infections risk: focus on herpes zoster. Ther. Adv. Musculoskelet. Dis. 12, 1759720x20936059. 10.1177/1759720X20936059 32655703 PMC7328488

[B136] Szwajkowska-Michałek L. Przybylska-Balcerek A. Rogoziński T. Stuper-Szablewska K. (2020). Phenolic compounds in trees and shrubs of central Europe. Appl. Sci. 10 (19), 6907. 10.3390/app10196907

[B137] Takano F. Takata T. Yoshihara A. Nakamura Y. Arima Y. Ohta T. (2007). Aqueous extract of peanut skin and its main constituent procyanidin A1 suppress serum IgE and IgG1 levels in mice-immunized with ovalbumin. Biol. Pharm. Bull. 30 (5), 922–927. 10.1248/bpb.30.922 17473436

[B138] Tan E. M. Smolen J. S. (2016). Historical observations contributing insights on etiopathogenesis of rheumatoid arthritis and role of rheumatoid factor. J. Exp. Med. 213 (10), 1937–1950. 10.1084/jem.20160792 27621417 PMC5030811

[B139] Tanabe S. Santos J. La V. D. Howell A. B. Grenier D. (2011). A-type cranberry proanthocyanidins inhibit the RANKL-dependent differentiation and function of human osteoclasts. Molecules 16 (3), 2365–2374. 10.3390/molecules16032365 21399573 PMC6259657

[B140] Terra X. Valls J. Vitrac X. Mérrillon J. M. Arola L. Ardèvol A. (2007). Grape-seed procyanidins act as antiinflammatory agents in endotoxin-stimulated RAW 264.7 macrophages by inhibiting NFkB signaling pathway. J. Agric. Food Chem. 55 (11), 4357–4365. 10.1021/jf0633185 17461594

[B141] Thomas T. Friedrich M. Rich-Griffin C. Pohin M. Agarwal D. Pakpoor J. (2024). A longitudinal single-cell atlas of anti-tumour necrosis factor treatment in inflammatory bowel disease. Nat. Immunol. 25 (11), 2152–2165. 10.1038/s41590-024-01994-8 39438660 PMC11519010

[B142] Thorleifsdottir R. H. Sigurdardottir S. L. Sigurgeirsson B. Olafsson J. H. Sigurdsson M. I. Petersen H. (2017). Patient-reported outcomes and clinical response in patients with moderate-to-severe plaque psoriasis treated with tonsillectomy: a randomized controlled trial. Acta Derm. Venereol. 97 (3), 340–345. 10.2340/00015555-2562 27819714

[B143] Tian Y. Mao X. Sun R. Zhang M. Xia Q. (2020). Enhanced oral bioavailability of oligomeric proanthocyanidins by a self-double-emulsifying drug delivery system. Food Sci. Nutr. 8 (7), 3814–3825. 10.1002/fsn3.1673 32724643 PMC7382205

[B144] Toda K. Tsukayama I. Nagasaki Y. Konoike Y. Tamenobu A. Ganeko N. (2020). Red-kerneled rice proanthocyanidin inhibits arachidonate 5-lipoxygenase and decreases psoriasis-like skin inflammation. Arch. Biochem. Biophys. 689, 108307. 10.1016/j.abb.2020.108307 32112739

[B145] Ungprasert P. Wijarnpreecha K. Wetter D. A. (2017). Periodontitis and risk of psoriasis: a systematic review and meta-analysis. J. Eur. Acad. Dermatol Venereol. 31 (5), 857–862. 10.1111/jdv.14051 27862342 PMC5408312

[B146] Valdimarsson H. Thorleifsdottir R. H. Sigurdardottir S. L. Gudjonsson J. E. Johnston A. (2009). Psoriasis--as an autoimmune disease caused by molecular mimicry. Trends Immunol. 30 (10), 494–501. 10.1016/j.it.2009.07.008 19781993

[B147] van Duynhoven J. Vaughan E. E. Jacobs D. M. Kemperman R. A. van Velzen E. J. Gross G. (2011). Metabolic fate of polyphenols in the human superorganism. Proc. Natl. Acad. Sci. U. S. A. 108 (1):4531–4538. 10.1073/pnas.1000098107 20615997 PMC3063601

[B148] Wang J. Yuan Z. Y. Wang X. Y. Zhu J. X. Huang W. F. Xu G. H. (2024). Anthocyanins-rich cranberry extract attenuates DSS-induced IBD in an intestinal flora independent manner. Curr. Res. Food Sci. 9, 100815. 10.1016/j.crfs.2024.100815 39161885 PMC11332073

[B149] Weichmann F. Rohdewald P. (2024). Pycnogenol(®) French maritime pine bark extract in randomized, double-blind, placebo-controlled human clinical studies. Front. Nutr. 11, 1389374. 10.3389/fnut.2024.1389374 38757130 PMC11096518

[B150] Weseler A. R. Bast A. (2017). Masquelier's grape seed extract: from basic flavonoid research to a well-characterized food supplement with health benefits. Nutr. J. 16 (1), 5. 10.1186/s12937-016-0218-1 28103873 PMC5248468

[B151] Williams J. P. Meyers J. A. (2002). Immune-Mediated Inflammatory Disorders (I.M.I.D.S): The Economic and Clinical Costs, 8. 12516953

[B152] Winchester R. Minevich G. Steshenko V. Kirby B. Kane D. Greenberg D. A. (2012). HLA associations reveal genetic heterogeneity in psoriatic arthritis and in the psoriasis phenotype. Arthritis Rheum. 64 (4), 1134–1144. 10.1002/art.33415 22006066

[B153] Wojdyło A. Oszmiański J. Laskowski P. (2008). Polyphenolic compounds and antioxidant activity of new and old apple varieties. J. Agric. Food Chem. 56 (15), 6520–6530. 10.1021/jf800510j 18611028

[B154] Xavier R. J. Podolsky D. K. (2007). Unravelling the pathogenesis of inflammatory bowel disease. Nature 448 (7152), 427–434. 10.1038/nature06005 17653185

[B155] Xiong D. K. Shi X. Han M. M. Zhang X. M. Wu N. N. Sheng X. Y. (2022). The regulatory mechanism and potential application of IL-23 in autoimmune diseases. Front. Pharmacol. 13, 982238. 10.3389/fphar.2022.982238 36176425 PMC9514453

[B156] Yan S. Kotschenreuther K. Deng S. Kofler D. M. (2022). Regulatory T cells in rheumatoid arthritis: functions, development, regulation, and therapeutic potential. Cell Mol. Life Sci. 79 (10), 533. 10.1007/s00018-022-04563-0 PMC9522664 36173485

[B157] Yang S. Wang J. Brand D. D. Zheng S. G. (2018). Role of TNF-TNF receptor 2 signal in regulatory T cells and its therapeutic implications. Front. Immunol. 9, 784. 10.3389/fimmu.2018.00784 29725328 PMC5916970

[B158] Yang B. Dong Y. Wang F. Zhang Y. (2020). Nanoformulations to enhance the bioavailability and physiological functions of polyphenols. Molecules 2022, 13. 10.3390/molecules25204613 33050462 PMC7587200

[B159] Yang Y. Zhao Y. Lai R. Xian L. Lei Q. Xu J. (2022). An emerging role of proanthocyanidins on psoriasis: evidence from a psoriasis-like mouse model. Oxid. Med. Cell Longev. 2022, 5800586. 10.1155/2022/5800586 35720176 PMC9200549

[B160] Yang J. Huang Q. Long J. Li J. (2025). Microbiota and inflammatory bowel disease: the dual effect mechanism of polysaccharide therapy. Front. Immunol. 16, 1666866. 10.3389/fimmu.2025.1666866 41246343 PMC12615394

[B161] Yin H. Liu N. Sigdel K. R. Duan L. (2022). Role of NLRP3 inflammasome in rheumatoid arthritis. Front. Immunol. 13, 931690. 10.3389/fimmu.2022.931690 35833125 PMC9271572

[B162] Yu A. Sun L. Chen L. Wang D. Li Z. Wang Y. (2025a). The biological functions of proanthocyanidin and its application in pig production. Front. Vet. Sci. 12, 1565501. 10.3389/fvets.2025.1565501 PMC11938249 40144517

[B163] Yu L. Feng S. Song Y. Bi J. Gao Y. Wang L. (2025b). Exploring the extraction, antioxidant activities and its stabilities of peanut skins crude proanthocyanidins extract. Sci. Rep. 15 (1), 26274. 10.1038/s41598-025-10934-x PMC12276230 40683947

[B164] Yuan Y. Mu N. Li Y. Gu J. Chu C. Yu X. (2025). TNF-α promotes synovial inflammation and cartilage bone destruction in rheumatoid arthritis *via* NF-κB/YY1/miR-103a-3p axis. Faseb J. 39 (14), e70876. 10.1096/fj.202501452r 40699912

[B165] Zeevi D. Korem T. Zmora N. Israeli D. Rothschild D. Weinberger A. (2015). Personalized nutrition by prediction of glycemic responses. Cell 163 (5), 1079–1094. 10.1016/j.cell.2015.11.001 26590418

[B166] Zeng Y. X. Wang S. Wei L. Cui Y. Y. Chen Y. H. (2020). Proanthocyanidins: components, pharmacokinetics and biomedical properties. Am. J. Chin. Med. 48 (4), 813–869. 10.1142/S0192415X2050041X 32536248

[B167] Zhang Y. Z. Li Y. Y. (2014). Inflammatory bowel disease: pathogenesis. World J. Gastroenterol. 20 (1), 91–99. 10.3748/wjg.v20.i1.91 24415861 PMC3886036

[B168] Zhang Y. Li M. Liu H. Fan Y. Liu H. H. (2025). The application of procyanidins in diabetes and its complications: a review of preclinical studies. Front. Pharmacol. 16, 1532246. 10.3389/fphar.2025.1532246 39995417 PMC11847907

[B169] Zhao Y. Xie Y. Li X. Song J. Guo M. Xian D. (2022). The protective effect of proanthocyanidins on the psoriasis-like cell models *via* PI3K/AKT and HO-1. Redox Rep. 27 (1), 200–211. 10.1080/13510002.2022.2123841 36178125 PMC9542435

[B170] Zhao Y. Jiang C. Lu J. Sun Y. Cui Y. (2023). Research progress of proanthocyanidins and anthocyanidins. Phytother. Res. 37 (6), 2552–2577. 10.1002/ptr.7850 37118988

[B171] Zhao X. Song Y. Zhao W. Shang L. Fan C. Xie J. (2025). CD8(+) T cells mediate the impact of gut dysbiosis and short-chain fatty acid deficiency on accelerated arthritis progression in collagen-induced arthritis mice. Front. Immunol. 16, 1702792. 10.3389/fimmu.2025.1702792 PMC12660273 41322410

[B172] Zhong L. Luo N. Zhong X. Xu T. Hao P. (2022). The immunoregulatory effects of natural products on psoriasis *via* its action on Th17 cells *versus* regulatory T cells balance. Int. Immunopharmacol. 110, 109032. 10.1016/j.intimp.2022.109032 35810491

[B173] Zhou D. Y. Du Q. Li R. R. Huang M. Zhang Q. Wei G. Z. (2011). Grape seed proanthocyanidin extract attenuates airway inflammation and hyperresponsiveness in a murine model of asthma by downregulating inducible nitric oxide synthase. Planta Med. 77 (14), 1575–1581. 10.1055/s-0030-1270957 21452107

[B174] Zhou D. Y. Fang S. R. Zou C. F. Zhang Q. Gu W. (2015). Proanthocyanidin from grape seed extract inhibits airway inflammation and remodeling in a murine model of chronic asthma. Nat. Prod. Commun. 10 (2), 257–262. 25920255

